# A multi-country study to co-design and evaluate digital educational resources to support conversations about ending fertility treatment

**DOI:** 10.1093/humrep/deaf248

**Published:** 2026-01-07

**Authors:** Mariana Sousa-Leite, Sofia Gameiro

**Affiliations:** School of Psychology, Cardiff University, Cardiff, UK; EPIUnit ITR, Institute of Public Health of the University of Porto, Porto, Portugal; School of Psychology, Cardiff University, Cardiff, UK

**Keywords:** unsuccessful fertility treatment, preventive end-of-treatment care, routine psychosocial care, co-designed workshops, digital educational resources, acceptability and feasibility

## Abstract

**STUDY QUESTION:**

How can educational resources be feasibly co-designed and used to support conversations between staff and patients about ending fertility treatment?

**SUMMARY ANSWER:**

Co-design workshops allow for the development of educational resources that account for all stakeholders’ perspectives and are considered sensitive, informative, and helpful to support end-of-treatment conversations, but staff and patients have different views about how these can be used within the treatment pathway.

**WHAT IS KNOWN ALREADY:**

Ending treatment without children is a common outcome but seldom discussed with patients. Preventive end-of-treatment care aims to promote healthy transitions at the end of treatment by preparing and helping patients cope with this possible outcome. Nine in ten patients want to receive such care, but only 3 in 10 report receiving it. Knowledge of perceived barriers to implementing preventive end-of-treatment care at clinics and whether digital educational resources can be developed to support its provision is lacking.

**STUDY DESIGN, SIZE, DURATION:**

Co-design workshops with fertility staff (March 2022), patients, and patient advocates (March–December 2022) from Europe (Belgium, Finland, Germany, Italy, Portugal, Spain, and UK) and South America (Argentina, Brazil, and Chile). Staff were invited to participate through fertility professional and scientific associations, and patients and advocates via charities and social media. Eligibility criteria were being aged 18 or older and working in fertility care (for staff) or charity (for advocates) or being waiting to initiate, undergoing, or having undergone treatment within 6 months (for patients).

**PARTICIPANTS/MATERIALS, SETTING, METHODS:**

A preliminary specification and initial prototypes of digital educational resources to support staff and patients, respectively, in having conversations about ending treatment were developed with relevant stakeholders. Co-design workshops with study participants were conducted. A semi-structured script, following Bowen *et al.*’s (2009)feasibility framework, was used to guide the workshops. Questions covered: (i) experiences, views, and preferences on the provision of preventive end-of-treatment care at clinics and iterative prototypes of the resources to support this provision (acceptability); (ii) perceived need and benefits (demand); and (iii) perceived barriers and facilitators to its implementation at clinics (practicalities). Workshops were recorded and transcribed verbatim, and data were analysed using Framework Analysis.

**MAIN RESULTS AND THE ROLE OF CHANCE:**

Fifteen fertility staff, 34 patients, and 7 advocates participated. Staff were mainly psychologists/counsellors (40.0%) or clinicians (26.7%) working in the field for around 23 years. Patients were mostly women (91.2%), on average aged 38 years. Most were childless (73.5%) and trying to conceive for around 3 years. Framework analysis of data collected during the co-design workshops generated four themes and one meta-theme, reflecting a need for a normative shift across countries towards the routine implementation of preventive end-of-treatment care. Themes reflected: (i) demand for routine provision of holistic psychosocial care, including preventive end-of-treatment care; (ii) different views between staff and patients about the risks and extent of benefits of routinely implementing preventive end-of-treatment care; (iii) patient high clarity about the functions of preventive end-of-treatment care (ensuring patients feel prepared and supported in moving through the grief and cope with short-term challenges; explore other pathways to parenthood and re-orient one’s life goals; and ensure informed consent for fertility treatment) versus staff lower clarity, with care being equated to signposting patients for timely psychological support; and (iv) co-designed digital educational resources are helpful to support the routine provision of preventive end-of-treatment care at clinics.

**LIMITATIONS, REASONS FOR CAUTION:**

Non-probability sample. Although the patient sample was heterogeneous (heterosexual and same-sex couples; private and public sectors), patients were primarily White, well-educated, employed, and childless women, limiting the generalization and comparisons across gender and other personal characteristics (ethnicity, socioeconomically disadvantaged, and disabled), where access to and acceptance of psychosocial support are expected to be lower.

**WIDER IMPLICATIONS OF THE FINDINGS:**

Routine discussions about the end of treatment are needed and beneficial, but staff will require reassurance and training on with whom, when, and how to engage in these. The final version of the digital educational resources is seen as valuable to support a cultural shift in implementing end-of-treatment preventive care at clinics. The co-designed webpages are freely available online in four languages (for staff: www.myjourney.pt/clinics, for patients: www.myjourney.pt/patients). Future research is needed to raise awareness and further investigate how best to support staff in such care provision and measure its impact.

**STUDY FUNDING/COMPETING INTEREST(S):**

This work was supported by a Research Wales Innovation Fund from the Higher Education Funding Council for Wales (HEFCW, grant No.: JA1710IF63). M.S.-L. was supported by the Portuguese Foundation for Science and Technology (FCT; fellowship No.: SFRH/BD/144429/2019) and the UK Economic and Social Research Council (ESRC; fellowship No.: ES/Z503125/1). The EPIUnit and ITR were funded by the FTC through the Portuguese State Budget (projects No.: UIDB/04750/2020 and LA/P/0064/2020 and DOI identifiers https://doi.org/10.54499/UIDB/04750/2020 and https://doi.org/10.54499/LA/P/0064/2020). S.G. reports grants from the European Society for Human Reproduction and Embryology (ESHRE), the Wellcome Fund (UK), and the Health and Care Research Wales (UK). Cardiff University holds the Intellectual Property rights for the tool www.myjourney.pt, licensed under a Creative Commons AttributionNonCommercial-ShareAlike 4.0 International Licence (CC BY-NCSA 4.0).

**TRIAL REGISTRATION NUMBER:**

n/a.

## Introduction

Many patients end fertility treatment without a live birth. In 2019, European cumulative estimations indicated that 81% of all ART cycles did not result in a live birth (Smeenk *et al.*, 2023). Even in countries where up to six IVF/ICSI cycles are funded, optimistic and conservative estimations show that 23 and 45% of patients, respectively, end all cycles without success (De Neubourg *et al.*, 2021). The negative and often long-lasting impact of the end of treatment is well-documented in the literature, showing it poses significant challenges for patients, including psychological difficulties, social isolation, and relational strain (Verhaak *et al.*, 2007; Gameiro and Finnigan, 2017). This impact could be mitigated if adequate psychosocial care were put in place (Kraaij *et al.*, 2016; Boivin *et al.*, 2022; Sousa-Leite *et al.*, 2022), which has been recommended by European guidelines and codes of practice (Gameiro *et al.*, 2015; HFEA, 2023). Some patients are signposted to psychosocial support, but it is unclear what proportion, under what criteria, and what the scope of the support provided is (Pasch *et al.*, 2016; Boivin *et al.*, 2022). While very few evidence-based interventions to support these patients exist (Kraaij *et al.*, 2016; Rowbottom *et al.*, 2022; Sousa-Leite, 2024), these are reported with different levels of detail, and only one is accessible for use by clinics or patients. This latter is MyJourney, a web-based, evidence-based psychosocial intervention aimed at supporting people who reach acceptance of their unfulfilled wish for children (Rowbottom *et al.*, 2022). However, anecdotal evidence suggests patients are not signposted to support (Payne and van den Akker, 2016; Fertility Europe, 2023), and recent survey research confirms patients report having few opportunities to discuss the implications of their treatment not resulting in childbirth, how to move forward, and what support they have available in that eventuality (Sousa-Leite *et al.*, 2023).

The authors have argued that adopting a preventive psychosocial approach to the end of unsuccessful treatment (hereafter referred to as preventive end-of-treatment care), whereby patients have the opportunity to discuss and develop insight about the implications of unsuccessful treatment while still doing treatment, may ease their adjustment when confronted with this outcome or help them recognize the need for and access additional support (Davis and Asliturk, 2011; Sousa-Leite *et al.*, 2023). Survey research indicates patients are receptive to preventive end-of-treatment care, with 9 in every 10 reporting they want this to be embedded in routine care offered at clinics (Sousa-Leite *et al.*, 2023). Some of the perceived benefits include coping better with unsuccessful treatment and making more informed and timely decisions about treatment and non-treatment options, both during and after treatment (Sousa-Leite *et al.*, 2023). These perceived benefits are supported by motivational and lifespan theories showing that people benefit from thinking about their capacity to continue pursuing one’s pathways to reach desired goals versus considering alternative pathways or adjusting their goals (Snyder, 2002; Su and Chen, 2006; Heckhausen *et al.*, 2010). Similar preventive approaches have proven helpful in other related health contexts (e.g. cancer treatment), where it was observed that providing patients with a comprehensive view of the benefits and potential adverse outcomes of treatment, common experiences, and appropriate coping strategies eased their adjustment when negative outcomes indeed occurred. These patients reported lower emotional distress and better well-being and quality of life than patients who did not receive such care (Thomas *et al.*, 2000; Waller *et al.*, 2014).

Recent studies, however, suggest that implementing preventive end-of-treatment care at fertility clinics can be challenging for both patients and staff. While patients recognize that discussing the possibility of treatment not being successful may trigger stress and impact hope for a positive outcome, they consider the benefits to outweigh these potential disadvantages (Sousa-Leite *et al.*, 2023). The views of staff towards preventive end-of-treatment care are less well known. Multicycle planning research suggests staff perceive similar risks in discussing negative treatment outcomes as patients do (e.g. deflating patient hope) but are more risk-averse (Harrison *et al.*, 2022). If preventive end-of-treatment care is to be implemented in clinics, a better understanding of staff concerns and how to address them effectively is critical. Together, this evidence suggests that more efforts should be put into exploring acceptable and feasible approaches to promote preventive end-of-treatment care at fertility clinics.

This study aimed to develop a further understanding of the factors that shape fertility staff’s and patients’ willingness to engage in preventive end-of-treatment care and co-design multilingual digital educational resources tailored to staff and patients to promote and support end-of-treatment discussions at fertility clinics. The work was informed by the Theory of Planned Behaviour (TPB; Ajzen, 1985) and the Health Belief Model (HBM; Rosenstock, 1974), as well as research on patient preferences about the format, delivery mode, and content of preventive end-of-treatment care (Sousa-Leite *et al.*, 2023), and user consultation. The TPB and HBM have been successfully used to inform the design of health education initiatives, having proven efficacious in predicting intentions or promoting and modifying preventive health behaviours in fertility care (e.g. reducing sexual-risk behaviours, enhancing fertility help-seeking; Fulford *et al.*, 2013; Tyson *et al.*, 2014). While evidence does not favour one model over another, the TPB was chosen to inform the staff resources, given its focus on procedural implementation aspects, and the HBM to inform the patient resources, as it is most often used with this population (Noar, 2005). A participatory design approach with multiple stakeholders (fertility staff, patients, and patient advocates) from Europe and Latin America was used to ensure the representation of end-users’ views, experiences, and preferences in the evolving prototypes being iteratively co-designed (Peters *et al.*, 2024). We considered three dimensions of acceptability that, according to Bowen *et al.* (2009), must be targeted when determining whether resources are appropriate and feasible for implementation: acceptability, demand, and practicalities. The specific goals of the study were to (i) investigate staff and patients’ experiences, views, and perceived need for preventive end-of-treatment care (i.e. acceptability and demand); (ii) co-design tailored digital educational resources to promote preventive end-of-treatment care; and (iii) investigate whether it is feasible to implement these resources at fertility clinics (i.e. practicalities).

## Materials and methods

### Design

Multi-country, iterative co-design workshop-based study.

### Participants

Eligible fertility staff were working in fertility care. Eligible patients were waiting to start or undergoing fertility treatment or had done it within the last 6 months. Eligible patient advocates worked at a fertility charity. Additional eligibility criteria for all participants included being an adult (aged 18 or older) and being able to read and speak English, Spanish, or Portuguese.

### Procedure

**Figure 1.**
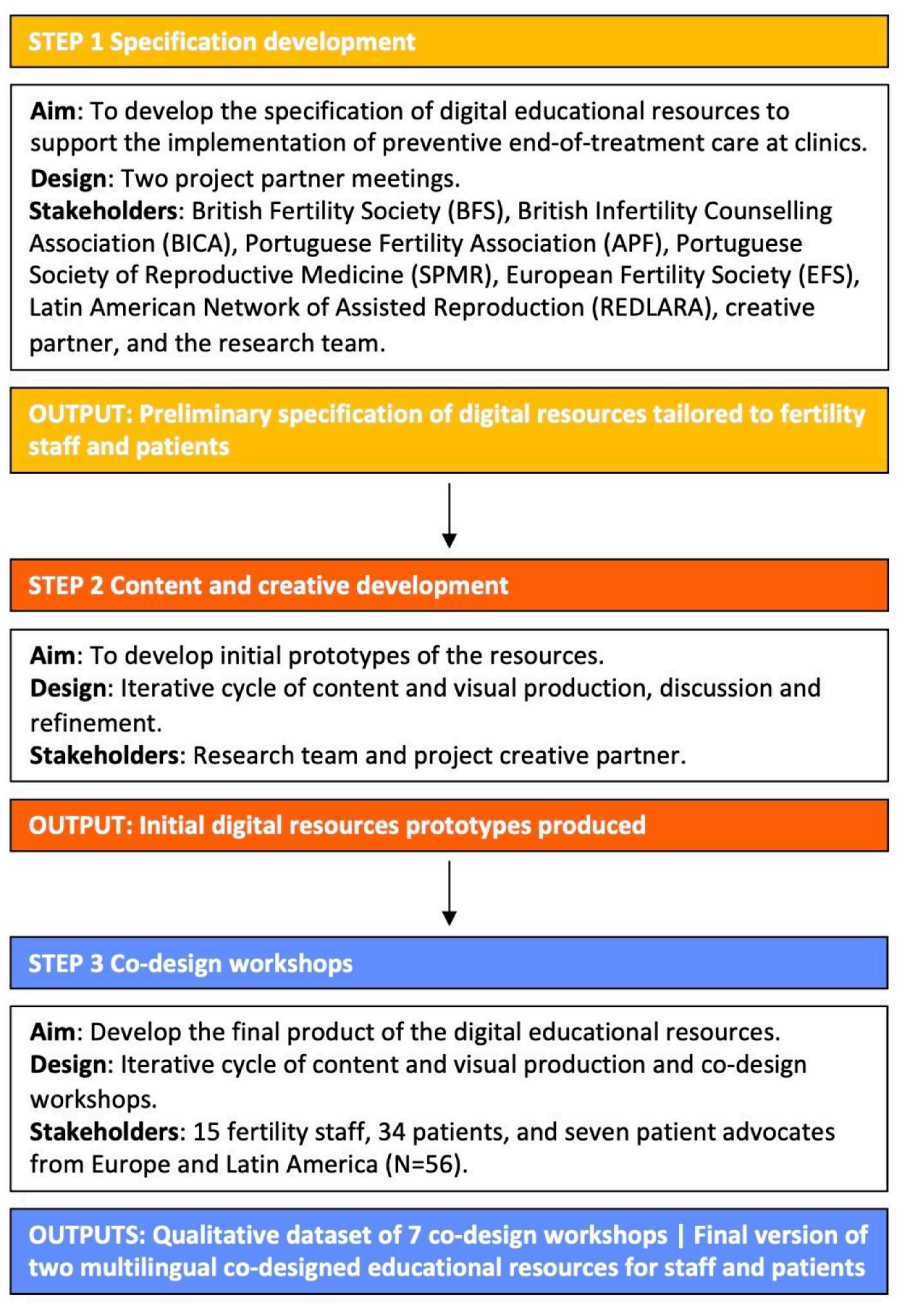
Development process for co-designing the multilingual digital educational resource.

Figure 1 presents an overview of the development process used to co-design the multilingual digital educational resources. Development was grounded in participatory design principles, emphasising the involvement of diverse stakeholders across all stages. We partnered with fertility scientific and professional societies and patient advocacy charities across Europe and Latin America to develop the specification of digital resources. These organizations represent the fertility stakeholders’ community and/or set care standards within the countries they represent.

#### Step 1—specification development

We conducted two project partner meetings with nine representatives from these fertility organizations and a creative partner to discuss how to introduce the concept of preventive end-of-treatment care and the media format, content, and delivery mode of the associated digital educational resources. The discussion was supported by a PowerPoint presentation (see Supplementary Data File S1), which contextualized the project and shared a high-level proposal for the resources created by the research team.

#### Step 2—content and creative development

The research team worked with the project’s creative partner over 3 months to develop initial prototypes of the digital resources (tailored to supporting staff and patients, respectively). This involved developing the educational content and its presentation in diverse digital assets using different media (e.g. text, images, animation, and flyers).

#### Step 3—co-design workshops

In this step, we engaged in an iterative cycle of eliciting feedback from research participants and prototype refinement. Specifically, we conducted five co-design workshops with the study participants to elicit their experiences, views, and preferences on the acceptability and feasibility of the resources’ prototypes. Subsequently, the prototypes were redesigned to integrate feedback received and presented for another round of acceptability and feasibility feedback in two additional workshops. Final versions of the resources were produced and translated into four languages (English, Spanish from Europe and Latin America, Portuguese, and German). Study participants were recruited via multiple pathways. A convenience sample of fertility staff members of leading fertility societies (e.g. European Society of Human Reproduction and Embryology [ESHRE] in Europe, British Infertility Counselling Association [BICA] in the UK, Portuguese Society for Reproductive Medicine [SPMR] in Portugal, and Latin America Scientific Society for Reproductive Medicine [REDLARA] in Latin America) were emailed (February–March 2022) an invitation to participate in the workshops (with a direct link to the information sheet and informed consent form). Patients and patient advocates were recruited (February–December 2022) via social media adverts (Facebook, Instagram, and Twitter), social influencers, and international fertility charities (e.g. Fertility Network UK [FNUK], Portuguese Fertility Association [APF] in Portugal, and Concebir Asociación Civil in Latin America). These latter were approached (via the use of a gatekeeper letter) and asked to distribute the study among their communities. UK patients were additionally recruited via Prolific, a well-established, trustworthy, and cost-effective online recruitment platform that allows researchers to invite a screened subgroup of people based on specific criteria (Peer *et al.*, 2017). As for staff, interested patients could click on the study link, which directed them to an information sheet and consent form. Participants who consented were automatically asked to report on their sociodemographic (all participants), professional (staff), and clinical (patients) characteristics (Qualtrics, Provo, UT, USA).

In accordance with best practice guidelines, the workshops were carried out online and separately for staff and patients to promote a safe and comfortable environment for participants to share their experiences and views without regard to the views of other groups (Hennink, 2014). Patient advocates were invited to attend the patients’ workshops to represent patients’ perspectives and encourage discussion. At the start, participants were provided with information about the study’s aims, procedures (including the recording, as per consent), and ground rules (e.g. confidentiality, welcoming all thoughts, even if negative or opposing, and freedom to ask additional questions). A semi-structured script was used to guide the discussion. Moderation promoted active and balanced engagement from all participants (Krueger and Casey, 2000). All workshops were conducted via Zoom (Zoom Video Communications), moderated/assisted by a clinical psychology-trained researcher (M.S.-L. and/or S.G.), had a 1 h/1 h 30 planned duration, and were video-recorded and transcribed verbatim. At the end, all participants were debriefed (with direct links to support resources), and patients were offered a £20 token for participation.

### Materials

#### Sociodemographic, professional, and clinical form

Participants were asked about their age, gender identity, and country of residence. Staff were also asked about their professional title (clinician, embryologist/andrologist, lab technician, nurse/midwife, psychologist/counsellor, clinic manager, other), workplace (public, private, both, other), and for how long they had been working in fertility care (in years). Patients and patient advocates were asked about their education and occupational status. Patients were additionally asked about their sexual orientation, relationship status and duration (if in a relationship, in years), parenthood status (no children, biological, adopted, stepchildren), whether they had children from fertility treatment (no, yes), their current situation regarding treatment (list of seven descriptors, e.g. undergoing diagnosis; other), and for how long they were trying or had tried to achieve a pregnancy or father a(nother) child (in years).

#### Co-design workshops script

Following existing guidelines (Krueger and Casey, 2000; Hennink, 2014), one semi-structured script comprising 14 open questions and informal clarification prompts was developed. The wording was adapted for each participant group (staff; patients and patient advocates; available in Supplementary Data File S2). The script started by defining ‘end of unsuccessful treatment’ as when all treatment cycles are unsuccessful, and no new cycles are being attempted in the future. Questions were informed by Bowen *et al.*’s (2009) feasibility framework and were organized into two sections. The first section targeted participants’ experiences and views of preventive end-of-treatment care provision at clinics (acceptability), perceived need for and benefits of such care (demand), and perceived barriers and facilitators to its provision (practicalities). The second section started by presenting participants with the prototype of the digital educational resources. Questions targeted participants’ views and first reactions to the resources, willingness to use them (acceptability), perceived positive effects and intention to use (demand), and barriers and facilitators to their provision at clinics (practicalities).

### Ethical approval

The Ethics Committees of the School of Psychology, Cardiff University (EC.21.11.09.6443G), approved the study.

### Data management and analysis

Descriptive statistics were used to describe the sample’s sociodemographic, professional, and clinical characteristics.

Framework analysis was applied to the qualitative data from the co-design workshops (Gale *et al.*, 2013), as this method provides an in-depth and holistic view of the data without losing the participants’ individual views and allowing for the differentiation of views between different stakeholder groups (staff and patients/patient advocates). All contributions are treated as data and analysed in the same way regardless of who provided them (Gale *et al.*, 2013). The verbatim transcripts were imported into NVivo software version 12 (QSR International). M.S.-L. and S.G. familiarized themselves with the audio recordings and transcripts. Using an inductive approach, M.S.-L. set codes (i.e. descriptive meaning labels) for each text segment. S.G. and M.S.-L. met several times to review the coding and disagreements on interpretation were discussed until consensus was achieved. Connections and differences across the codes were analysed and systematically organized into categories. The main categories were then organized into themes (i.e. interpretative descriptions of several categories describing interrelated ideas) and one meta-theme. A data matrix was created, with the categories and themes in different rows, stakeholders’ groups (staff; patients and patient advocates) in columns, and a summary of the codes along with supporting representative verbatim quotes (translated into English) in the cells. ‘(…)’ indicates that part of the quote was omitted as it did not add relevant information, and ‘[text]’ represents clarifications added by the authors. W, workshop; Pa, patient; Adv, patient advocate; CL, clinician; EMB, embryologist/andrologist; N, nurse/midwife; Psych, psychologist; CM, clinic manager; ETH, ethicist.

## Results

### Step 1—specification development

The output of this first development step was a preliminary specification for two webpages, one tailored to fertility staff and another to patients, which can be found in the Supplementary Data File S3. Stakeholders agreed that webpages were the optimal format for the resources and that these should be hosted within the MyJourney online web app (www.myjourney.pt; Rowbottom *et al.*, 2022) to create a hub for end-of-treatment information.

### Step 2—content and creative development

Based on the agreed specification, two webpages were created. The webpage for staff aimed to strengthen their intentions to engage in end-of-treatment conversations with patients. Guided by the TPB, the webpage provided information on patients’ high willingness to receive preventive end-of-treatment care due to its multiple perceived benefits (positive attitudes), recommendations from fertility regulators and international guidelines to support patients adjusting to unsuccessful treatment (norms), and to build skills on how to provide preventive end-of-treatment care according to patients’ expressed preferences (perceived behavioural control; Sousa-Leite *et al.*, 2023). The webpage for patients provided information about the end of treatment and associated coping resources. Informed by the HBM, this webpage embedded a short video animation introducing the possibility of treatment not working (perceived susceptibility), followed by research-informed information on common patient experiences in the aftermath of this outcome (perceived severity), barriers and benefits of engaging in preventive end-of-treatment care, and emotional and coping resources, including signposting to MyJourney online web app. Both webpages included answers to frequently asked questions and concerns fertility patients raise on this topic (Gameiro and Finnigan, 2017).

### Step 3—co-design workshops

#### Participants

Two co-design workshops (March 2022) with 15 fertility staff and five workshops (March–December 2022) with 34 patients and 7 patient advocates were conducted. Supplementary Tables S1 and S2 present the composition of each workshop and participants’ characteristics. Staff were, on average, aged 51 years (*SD *= 13.65, range [32–75]). Most were women (n = 12, 80.0%), from Europe (n = 11, 73.3%) or South America (n = 4, 26.7%), and working in the private (n = 10, 66.7%) and public (n = 6, 40.0%) sectors, with an average of 23 years of work experience in the field (*SD *= 13.10, range [10.00–49.00]). Professional roles included psychologists/counsellors (n = 6, 40.0%), clinicians (n = 4, 26.7%), nurses/midwives (n = 2, 13.33%), an embryologist/andrologist (n = 1, 6.67%), a clinical manager (n = 1, 6.67%), and an ethicist (n = 1, 6.67%). Patients were, on average, aged 38 years (*SD *= 3.75, range: 30.00–44.00). Most were women (n = 31, 91.2%), from South America (n = 23, 69.7%) or Europe (n = 10, 30.3%). Most had a university education (n = 27, 79.4%) and were employed (n = 30, 90.9). Most self-identified as heterosexual (n = 26, 76.5%) and were in a relationship (n = 32, 94.1%) for around 10 years (*SD *= 4.62, range [1.00–17.17]), with a minority having biological children (n = 4, 11.76%) or stepchildren (n = 5, 14.71%). On average, patients were undergoing treatment for around 3 years (*SD *= 2.43, range [0.42–9.00]), with similar proportions waiting to start a(nother) cycle of treatment (n = 12, 35.3%), undergoing a cycle (n = 10, 29.4%), and the remaining third (n = 12, 35.3%) having finished treatment within the past 6 months. Patient advocates were, on average, aged 45 years (*SD *= 12.56, range: 35.00–64.00). Most were women (n = 6, 85.7%), from Europe (n = 4, 57.1%) or South America (n = 3, 42.9%). All had university education and were employed.

#### Thematic themes

**Figure 2.**
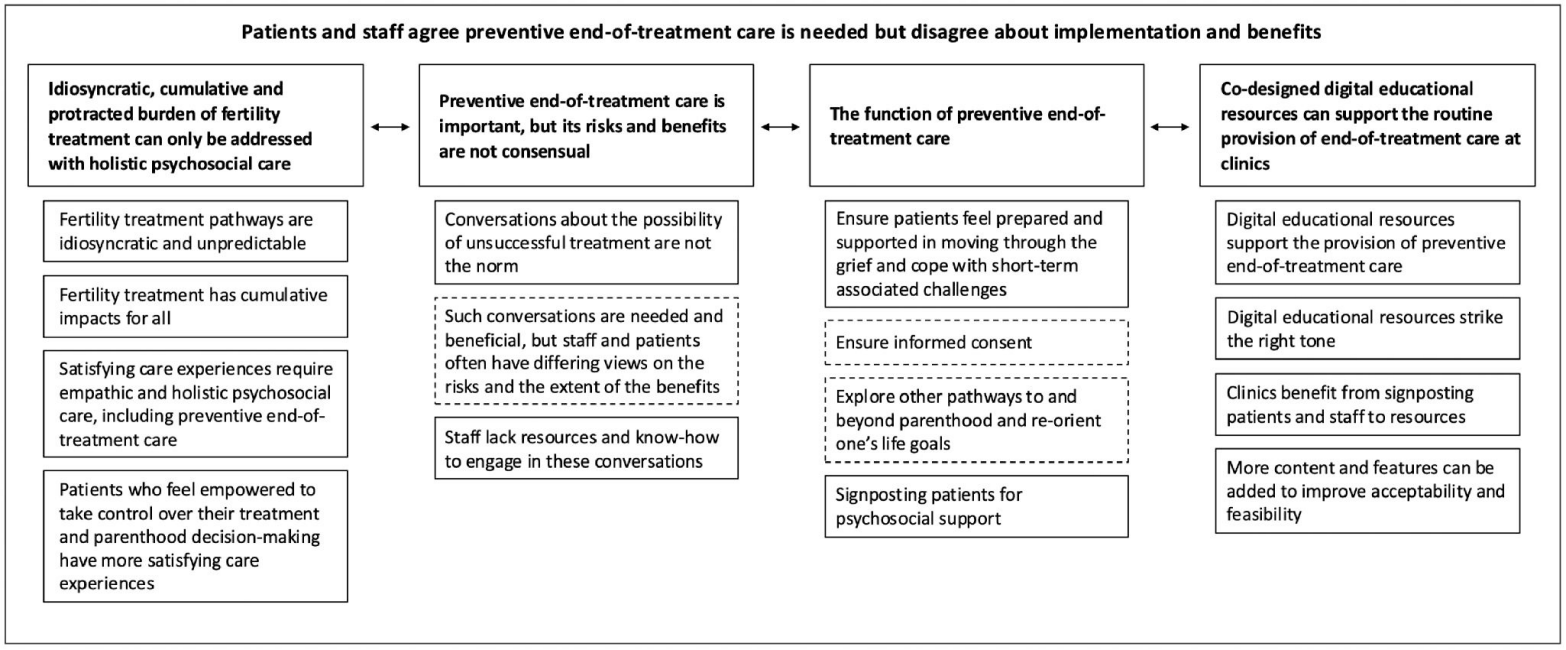
**Framework thematic map.** Fifteen categories grouped into four themes and one meta-theme. Continuous lines represent consensus between staff and patients/patient advocates, and dashed lines represent some level of disagreement.

Framework analysis yielded 650 codes, systematically organized into 15 categories, 4 themes, and 1 meta-theme. Figure 2 depicts the framework thematic map, and Supplementary Table S3 presents the data matrix. All themes and categories of codes were endorsed by staff and patients/patient advocates. Some categories were more endorsed by one group of participants than the other, and some reflected different views. Overall, when differences in experiences were reported within the same stakeholder group, this variation appeared to be associated with experiences in the private vs public healthcare sectors and did not seem to be related to the country of residence.

##### Meta-theme: patients and staff agree preventive end-of-treatment care is needed but disagree about implementation and benefits

Staff and patients agreed that better integration and continuity of multidisciplinary, holistic, and empathic psychosocial care throughout the whole treatment pathway (before, during, and after treatment) is needed at fertility clinics and that this should include preventive end-of-treatment care. Patients highlighted that preventive end-of-treatment care is not the norm and that more attention should be put into its routine provision as part of informed consent. Patients were clear about what should be the aims of preventive end-of-treatment care: ensuring they feel prepared and supported in moving through the grief, coping with the implications of unsuccessful treatment, and exploring other pathways to parenthood or re-orienting their life goals beyond parenthood. Staff seemed less certain about the aims of preventive end-of-treatment care and saw it mostly as signposting patients for specialist psychological support, considering that psychologists and counsellors would be better equipped to approach the possibility of treatment not working with patients. Staff thought providing preventive end-of-treatment care during the early stages of treatment was risky because most patients would not be ready to consider that treatment may not work, and such conversations are likely to trigger distress. Staff also stressed they feel unprepared to provide preventive care, lacking the required knowledge on when and how to offer it to their patients, as well as resources to support its provision. Both patients and staff evaluated the co-produced digital educational resources positively, seeing these as acceptable and feasible to use in clinics as an adjunct to facilitate the provision of end-of-treatment care. Several suggestions were made to optimize the resources.

##### Theme: idiosyncratic, cumulative, and protracted burden of fertility treatment can only be addressed with holistic psychosocial care

Staff and patients talked about how individual treatment trajectories are highly idiosyncratic and difficult to predict. Idiosyncrasy results from the diverse reasons patients have to seek treatment (e.g. health reasons, same-sex couples), the legal and logistic variations in access to treatment (e.g. donation, private sector), and individual treatment responses (‘*each of them [treatment cycle] failed at a different stage*’, W5, Pa1) and experiences (‘*we’re not entirely sure that we will stop, but we have definitely stopped with our current clinic, partly because of how they handled this*’, W5, Pa2). For patients, the unpredictability of their treatment journey results from the low success rates, lack of control over treatment outcomes, repeated and many times unexpected complications, unsuccessful cycles and losses, and the protracted nature of treatment caused by long waiting lists, and providers often offering new treatment options and add-ons. Regarding these latter, both staff and patients acknowledged the high financial costs of treatment and the business model of ART and the for-profit reproductive medicine market. Patients endorsed these factors as sources of psychological burden more than staff did and stressed additional ones that, in their view, were the most impactful and could be better managed at clinics: the lack of psychosocial care and forewarning and preparation for treatment complications and adverse outcomes (‘*My first cycle ended like a surprise ectopic [pregnancy], and I definitely did not feel prepared for the idea that there were other outcomes besides pregnant or not pregnant, and that was a really, really huge shock*’, W5, Pa2), including the end of treatment, which was described as difficult to accept and triggering unexpected feelings of denial, anger, and frustration.

*I never expected to respond the way I did [when treatment was unsuccessful] (…) my world fell through the floor when I got the news, and I’ve never known a feeling like it, and it is the most isolating thing in the world even if you were doing it as a couple*. (W5, Pa1)

Patients and staff agreed on multiple aspects of care that contribute to (dis)satisfying experiences of care. These mapped into three areas of patient-centred care that were more strongly endorsed by patients: empathic care, organization of care (personalization), and shared decision-making. Both groups valued empathic, timely, and responsive care during and immediately after challenging key treatment procedures and in situations of stress and loss. This included providing empathic preventive end-of-treatment care according to the patient’s values, preferences, and level of readiness (‘*needs to be considered, it needs to be respectful, and it needs to be given the time that it deserves*’, W4, adv2). However, many patients perceived a lack of empathic communication skills from staff, particularly when discussing adverse outcomes (‘*I started crying during the phone call [to inform about an unsuccessful cycle], and there wasn’t even nearly a validation of my feelings*’, W3, Pa1). Most felt they were treated as in a ‘*conveyor belt*’ (W5, Pa1, Pa5), as care was provided in a rush and ‘*support was totally deficient*’ (W2, Pa1), particularly after unsuccessful cycles or treatment: ‘*they just left us*’ (W5, Pa1). Experiences of shared decision-making contributed to a higher perception of patient-centred care and patients’ overall satisfaction. Positive determinants were related to having time to discuss and ‘*answer all our questions*’ (W2, Pa8) and discuss all treatment-related options. However, most patients reported not being involved in decision-making about their treatment plan and not receiving important information, in particular about unsuccessful treatment outcomes, to make informed decisions about treatment and parenthood. Patients had to be proactive in doing their ‘*own research*’ (W4, Pa1), as by default, information would not be provided, and many were unsure they could trust the information they accessed (mainly online). Patients also argued that fertility care, including end-of-treatment care, should address medical and psychosocial needs and requires involvement of all staff members (‘*the doctor, the psychologist, and the endocrinologist*’, W2, Pa4), but that currently there is ‘*no articulation between psychologists and clinicians*’ (W1, Pa2), the care ‘*was very medically oriented, professionals [clinicians] didn’t have training on the psychological impact of treatment*’ (W5, Pa4), and there was no referral for support, meaning ‘*the patient has to ask for an appointment*’ (W1, Psych1). Patients and staff agreed that the private sector has more resources to invest in patient psychosocial and personalized care, but even so, current provisions are insufficient.

##### Theme: preventive end-of-treatment care is important, but its risks and benefits are not consensual

Patients expressed more attention should be paid to forewarning and preparing them for possible negative outcomes, including the end of unsuccessful treatment (‘*I do feel a great need to prepare for the possibility that nothing works or that each [cycle] won’t work*’, W2, Pa11). However, both staff and patients reported that these conversations are not the norm (‘*we often don’t have that conversation*’, W1, N1). Patients reported that ‘*only the treatment success chances*’ (W4, Pa2) are mentioned, with most treatment-related discussions being focused on ‘*what the next [treatment] step will be*’ (W3, Pa1). *The possibility of ‘stopping trying is never mentioned*’ (W2, Pa13), and alternative (parenthood) pathways, such as adoption, ‘*aren’t mentioned either*’ (W1, Pa6). Three patients reported they discussed this possibility with their psychologist, considering it challenging but very helpful (‘*with the psychologist, we have worked a lot that we really realise that there is a life without children, that you can also be happy, that you can do a bunch of things*’, W2, Pa10). The exception is gamete/embryos donation, which is discussed but only towards the end of the treatment process and as the last resort.

*I remember asking the doctor in one of the appointments what would happen, so what the next [treatment] step would be if it [the treatment cycle] didn’t work, and he even said to me: -oh, let’s not think about it now, like, let’s be optimistic*. (W3, Pa1)

Clinic staff stated they often inform patients about treatment success rates but highlighted that patients ‘*don’t internalise these, they always think: —okay, it may not work, but it’s going to work for me*’ (W1, Psych4). They reported only discussing the possibility of treatment not working with ‘*very, very, very few patients*’ (W1, CL1), specifically those with very poor prognosis or those who are ‘*certain they will not have more treatment, with you nor anywhere else*’ (W1, N1). Although staff verbalized the importance of having such conversations, these were seen as risky and with unclear benefits. Staff were particularly worried that starting such conversations would be inappropriate given that patients tend to be very invested in and hopeful about treatment, not being emotionally prepared to discuss the possibility of a negative outcome (‘*if you concentrate on what cannot be done with treatment (…) when you stop the treatment, then I can see how the couple disintegrates because they want to explore other possibilities, other clinics abroad or anywhere else (…)*’, W1, CL1). Staff also expressed concerns that conversations about the end of treatment could trigger negative emotions that are difficult to manage and dissatisfaction towards the clinic (‘*they would be extremely angry*’, W1, CL1). In this context, staff reported being reluctant to label a cycle as the ‘*last one*’ (W1, Psych4) due to the difficulty of knowing when treatment really ends for each patient (‘*the difficulties are reaching the end of the road, rather than being at the end of the road*’, W2, CL1). Patients also recognized such ‘*conversations are hard*’ (W4, adv2) and that there should be a ‘*delicate balance*’ (W5, Pa2) between realism and hope, as patients need ‘*the energy and the hope, you know, to get through an incredibly difficult journey*’ (W4, adv2). Patients thought staff should tailor preventive end-of-treatment care according to each patient profile. However, patients perceived these conversations to have worthwhile benefits, referring to ‘*not having any conversation at all about the impact of it failing would lead to bigger trauma then, if experienced*’ (W5, Pa1).

Associated with these concerns, both patients and staff highlighted the lack of resources to support end-of-treatment conversations (‘*there is so much in the whole world. But this, the aftermath [of unsuccessful treatment], there is nothing. If you search for it, there’s nothing*’, W2, adv1). Staff felt unprepared, not knowing what information should be provided, how it should be provided, and when (‘*it is a difficult job*’, W1, CL1; ‘*the majority of the doctors are not trained right from the beginning to approach this topic with their patients*’, W2, CL2). Overall, staff seemed unsure about what type of support they can realistically offer patients in preparation for and when treatment does not work.

*many clinics don’t have that conversation early enough because they don’t have anything to really offer*. (W1, N1)

##### Theme: the function of preventive end-of-treatment care

This theme captured the challenges of ending treatment without achieving the desired child(ren) and the functions preventive end-of-treatment care should serve. Patients were certain that being informed and prepared for the possibility of treatment not working would make them trust their fertility clinic more and help them to ‘*have more knowledge*’ (W1, Pa5), ‘*not being given false expectations*’ (W2, Pa2), ‘*receive more psychological support*’ (W1, Pa5), ‘*make more informed decisions*’ (W5, Pa1), and have the ‘*confidence*’ (W1, Pa1, Pa5) and ‘*the tools to face those times when treatment fails*’ (W1, Pa1). Patients reported that preventive end-of-treatment care should be a way to receive information about ‘*what you could do in those circumstances to cope with feelings of grief*’ (W5, adv1) and how to manage relational and social relationships and different types of support, in particular, psychosocial (group) support. Medical information must include individual prognosis, ‘*good explanation of all steps of treatment*’ (W3, Pa4) and ‘*what can go wrong at each step*’ (W2, Pa2), ‘*how many rounds of treatment*’ (W4, adv2) and ‘*all [treatment] options*’ (W2, Pa11). Patients also referred that they would like to discuss which and how to access alternative pathways to and some beyond parenthood (including adoption and childfree lifestyle; ‘*so, what’s plan B? If plan A doesn’t work, what will be plan B, or plan C or plan D?*’, W3, adv1). Patients highlighted these conversations should be informative, ‘*open and honest*’ (W4, adv2), and offered to all patients, as they are ‘*vitally important at the beginning before treatment commences*’ (W4, adv2) and they ‘*are part of informed consent*’ (W2, Pa1). Some patients stressed the possibility of revisiting these conversations throughout treatment and that the approach and depth of such conversations should be tailored to the patient’s needs and preferences.

*It’s my right to be informed from the beginning, perhaps that I am going to undergo treatment and how these things can happen, and then I give consent (…) assuming those risks and knowing*. (W2, adv2)

Staff were concerned about how in-depth end-of-treatment conversations could be without superimposing the hope for a successful treatment. Staff envisioned end-of-treatment care as a means to provide information about the likelihood of successful and unsuccessful treatment, discuss the uptake of treatment cycles, and signpost for psychological support. Only two members of staff mentioned the importance of addressing alternative routes to parenthood, but when they did, other staff tended to concur. Staff referred that they would feel more comfortable providing preventive end-of-treatment care if they had support sources to signpost patients to.

*If you’re among those 30 unlucky per cent, then we’ll also, you know, offer you some support to go on with your life. I think that would be like a really important thing to offer, and it could even help us (…), because we might be brave enough to say that to the patient because we can offer them some support afterwards… because it’s all interconnected*. (W1, N1)

Although staff recognized patients would benefit from preventive end-of-treatment care from the start, they considered actual preparation and planning for the end of treatment would be difficult while pursuing treatment due to patients’ lack of willingness and readiness.

*because I oftentimes think in the beginning, patients are given a lot of hope. You don’t want to say: well, you know, it’ll probably not work, but we’ll try to treat you. So, you’ll say—We’ll do everything we can to help you*. (W2, ETH1)

##### Theme: co-designed digital educational resources can support the routine provision of preventive end-of-treatment care at clinics

All participants agreed that the co-produced digital educational resources can support the provision of preventive end-of-treatment care at clinics. All expressed very positive views towards the digital educational resources, considering these ‘*reliable*’ (W3, Pa1), ‘*super-interesting*’ (W2, adv2), and ‘*very useful*’ (W1, Psych4). All patients were highly willing to engage with the resources (‘*without a doubt that [after the clinical appointment] I would be curious to explore these better at home, in a private and safe place*’, W3, Pa1). Staff also referred that most patients ‘*would definitely want to explore that in their own surroundings and time*’ (W1, Psych2) and that they would be willing to offer these to their patients. However, they expressed concerns about exploring these in the consultation due to lack of time, appropriateness, and adequate training (‘*how can this project fit in reality? When clinicians are with a patient in front of them, how would they share this information?*’, W2, Psych1).

Patients perceived more benefits from resources (‘*support, information, guidance, points for reflection, ways that you can try and progress and move forward*’, W5, Pa5) than staff, but, overall, both agreed these are beneficial to support patients (‘*it’s comforting (…) it’s like a virtual hand, isn’t it!?*’, W3, Pa2) and a valuable training tool ‘*not only for the clinicians but for all clinic staff*’ (W2, CL3). Although some patients agreed the resources can trigger negative emotions and impact their engagement with treatment, patients believed that, as the resources included ‘*signposting links and contacts they [patients] can seek for further advice or support*’ (W5, Pa5), they would find it supportive and comforting (‘*I think that if this type of information reached everyone on time, even if it’s cruel, it would avoid a lot of pain*’, W2, Pa9).

*I really needed something like that, some support like that (…) it’s very valuable for patients*. (W1, Pa1)

All participants considered that the resources strike the right tone. They appreciated the resources being self-administered, online, and ‘*open and free*’ (W1, Pa1). All participants appreciated the ‘*mixed media. I like that you have the video and then you have the written part*’ (W1, N1), considering ‘*the questions [common questions and concerns] and the video very, very well done*’ (W1, Pa5) and ‘*brilliant*’ (W4, adv2). Aligned with the experiences reported above, patients considered that the resources should be disseminated as much and as early as possible and that all staff should be involved in their dissemination at the clinics. In particular, patients referred that these resources should be embedded in the clinics’ website, with many patients stressing they would be much more likely to choose a clinic presenting this information (‘*I would say that this clinic would immediately go up a few points in my consideration (…) [It] would demonstrate the clinic or the hospital is concerned with the emotional part of the treatment*’, W3, Pa1,—‘*Me too*’, W3, Pa4). Two patients referred that they may would like to be signposted to the resources and have more in-depth discussions with staff after at least one unsuccessful cycle because ‘*at another time, it could be a hard blow*’ (W5, Pa3). In contrast, most staff considered these resources ‘*should come later*’ (W1, N2, Psych3) in the treatment pathway. Some suggested signposting patients during the ‘*cycle review appointment*’ (W1, Psych2) after at least one cycle ‘*had completely failed*’ (W1, N1), as patients would more easily ‘*relate*’ (W1, N1) with the resources after having experienced one unsuccessful cycle. Most staff also agreed the resources could be made available on the clinic’s website ‘*but not on the front page, it will be down at the bottom, somewhere*’ (W1, N1), ‘*like additional information*’ (W1, CL1), as they were concerned they could ‘*scare patients away*’ (W1, CL1) and negatively impact their trust in the clinic. All participants provided suggestions on further content and features that could be included to improve the resources’ usefulness, specifically, further emotional and coping resources, support links and testimonies, higher personalization, and tailoring to underserved groups (e.g. men, same-sex couples, minoritized cultures).

#### Final version of two multilingual co-designed educational resources for staff and patients

A final version of educational resources was developed that embedded the workshop participants’ feedback. The webpage for staff included an additional section with materials that staff can print and use to ease the provision of end-of-treatment preventive care (e.g. information flyers, posters). The patients’ webpage included a new section focusing on alternative routes to parenthood, with signposting to different organizations (gamete and embryo donation, surrogacy, adoption, and fostering). The resources are now freely available online for public use in four languages: English, Spanish (from Europe and Latin America), Portuguese, and German. The staff and patients’ webpages can be accessed at www.myjourney.pt/clinics and www.myjourney.pt/patients.

## Discussion

This study highlights a gap between high patient demand for preventive end-of-treatment care and staff reluctance to offer it. The possibility that treatment may not work is rarely discussed in clinics, but patients find such conversations necessary and even protective. Results indicate it is possible to develop acceptable resources to support conversations about ending treatment. The resources developed were well-received for delivering sensitive, accurate, and accessible support. There was consensus among staff and patients about their value in facilitating conversations, supporting patient coping, and promoting informed decision-making. Nonetheless, additional measures are needed to shift clinical norms and address staff concerns and knowledge gaps. Further research, training, and interventions should explore how to implement preventive end-of-treatment care in a hopeful and supportive way and evaluate its impact on staff and patient outcomes.

The contrast between high demand and lack of access to preventive end-of-treatment validates previous research showing that treatment-related discussions tend to focus on the ‘next treatment step’ and achieving a successful outcome (Peddie *et al.*, 2004, 2005; Harrison *et al.*, 2021, 2022; Sousa-Leite *et al.*, 2022, 2023), despite patients expressing a need and willingness to discuss adverse outcomes (Dancet *et al.*, 2010; Harrison *et al.*, 2021, 2022), including ultimate failure (Sousa-Leite *et al.*, 2023). Staff’s reluctance to engage in end-of-treatment conversations and gatekeep provision of potentially distressful information (and its timing) reflects concerns about patient safety. However, this seems to trigger patient dissatisfaction, confusion, and feelings of deception and disempowerment (Fallowfield *et al.*, 2002; Brighton and Bristowe, 2016). This contradicts patient-centred care models that call for collaborative decision-making and full information sharing (van Empel *et al.*, 2008; Barry and Edgman-Levitan, 2012). While we argue that patients should be informed early about the possibility of treatment not working, we acknowledge the challenges of determining when and how to do this and the need for further research to evaluate the pros and cons of different approaches (timing, delivery—who, how, which content, format). Our data revealed divergent agendas between staff and patients. Staff reported concerns that early discussions about the possibility of treatment not working negatively impact patients’ engagement with treatment. While this should be carefully balanced considering the cumulative higher success rates of undergoing multiple treatment cycles, evidence suggests these discussions are unlikely to impact patients’ engagement with treatment. Patients indicated that with the appropriate psychosocial support and clear communication, they would feel more reassured, better supported, and trust the clinic more. This aligns with wider evidence showing that transparent information provision can improve patient satisfaction and coping rather than discourage continued treatment (Hammarberg *et al.*, 2001; Dancet *et al.*, 2010). Moreover, systematic reviews indicate that only a minority of patients (<10%) discontinue treatment because of reduced hope in treatment success or poor prognosis, as the majority discontinue for other reasons, such as the psychological burden of treatment, relational and personal problems, organizational barriers, and financial constraints (Gameiro *et al.*, 2012). Staff’s concerns that such conversations trigger anxiety in patients are valid, but this is a normative patient reaction that occurs in many information delivery contexts (e.g. single-cycle success rates, explanations of oocyte collection procedures; Devroe *et al.*, 2022; Dias *et al.*, 2025). The field can learn from other health fields (e.g. oncology, end-of-life care) where similar concerns have been overcome, and research has proved preventive care to be beneficial in promoting patients’ psychosocial adjustment in the face of adversity (Fallowfield *et al.*, 2002; Lyon *et al.*, 2014), leading to its integration in evidence-based best practice recommendations (Ngo-Metzger *et al.*, 2008; General Medical Council, 2010; NHS Improving Quality, 2014). To illustrate, research in oncology and palliative care has addressed staff’s concerns about causing despair or loss of hope by showing that providing honesty enables a greater sense of control and end-of-life planning, which is often linked to hope (Clayton *et al.*, 2005). These studies further highlight that empathic and patient-centred communication is crucial for maintaining trust and hope (Leone *et al.*, 2017). By integrating these lessons, we argue that resources for ending fertility treatment should be designed not only to provide clear information but also to ensure that such communication is delivered in a supportive and empathetic manner. Indeed, according to patients in this study, staff’s concern that end-of-treatment conversations may trigger dissatisfaction seems unfounded. Interview-based studies suggest this concern may reflect beliefs that their duty of care relies on treatment being successful and their own feelings of ‘helplessness’ and ‘debasement’ when confronted with the limits of assisted reproduction (Meier *et al.*, 2001; Fedele *et al.*, 2020). An additional point of consideration is the business model of ART, as mentioned by both patients and staff. The authors acknowledge that the for-profit reproductive medicine market of ART needs to be considered, and its role in communication about treatment outcomes further explored (Patrizio *et al.*, 2022).

While the views of patients in this study were similar across the different regulatory and public funding contexts considered (Europe, South America), these could vary for other contexts such as the USA, where IVF is not reimbursed and the average out-of-pocket costs per IVF cycle are three times higher than what patients are willing to pay (Murali *et al.*, 2025). Indeed, multiple evidence from psychological studies show that the more people invest their resources (emotional, physical, financially) in achieving an outcome, the more likely they are to disregard negative information or risk of negative outcomes (e.g. sunk cost effect, motivated reasoning, loss aversion; Kunda, 1990; Tom *et al.*, 2007; Sweis *et al.*, 2018). This indicates that the acceptability of end-of-treatment care may vary with investment context (amount spent, number of cycles done, financial resources spent), or it may need to be framed differently. For instance, research indicates that if psychological distance is increased (e.g. ‘would you advise a friend to use these materials’) or the framing is changed (e.g. ‘gaining information’ instead of ‘coping if treatment does not work’), people will be more likely to consider negative outcomes (Zikmund-Fisher *et al.*, 2006; Gallagher and Updegraff, 2012). Notwithstanding, overall results are reassuring in showing that, as seen in other fertility and health contexts, patients’ satisfaction and perceived quality of care are determined by the quality of the relationship with staff, information received, particularly about treatment burdens and adverse outcomes, and support received while navigating such outcomes (Leung *et al.*, 2012; You *et al.*, 2014).

Indeed, from the patients’ perspective, preventive end-of-treatment care should serve three functions: (i) ensuring informed consent by providing a full overview of the treatment pathway, including treatment options and potential negative outcomes (unsuccess, complications), (ii) supporting coping with loss and grief, and (iii) helping explore alternative avenues to and beyond parenthood. Optimal delivery may require a phased approach, prioritizing informed consent at the start, and coping and planning support towards the end of patients’ journey, or when patients demonstrate readiness to explore alternative scenarios (Heckhausen *et al.*, 2010; Rowbottom *et al.*, 2022). A challenge here is predicting the end of patients’ treatment journey, given the idiosyncrasy reported and the factors that contribute to patients’ readiness to receive preventive end-of-treatment care. Timing is a valid consideration, but data show that even when cycles are fully funded, 26.5% and 29.4% of patients end treatment after the first and second cycles, respectively (De Neubourg *et al.*, 2021). Behavioural change models, such as those used in this study or others (e.g. Transtheoretical Model of Change; Prochaska and Velicer, 1997), can be used to specifically investigate readiness and (its) visible markers, which staff can use to decide when to approach end-of-treatment care. A recent qualitative study showed that women navigate specific cognitive reframing strategies when considering stopping treatment (Bluth, 2023). The study suggests that women are open to considering end-of-treatment when they develop broader views of what their future may look like (i.e. not only with genetical/biological children), reflect on the risks and chances of achieving genetical/biological children compared to other parenthood (and non-parenthood) outcomes, and reconnect with and revaluing their body and mind. The implications are that staff need to engage in discussion with patients to elicit this information and support women navigating these cognitive processes, ensuring that ending treatment is seen as an empowering outcome rather than a failure. However, there remains a critical gap in understanding which end-of-treatment outcomes are valued by staff. Future research should explore staff’s understanding of what constitutes successful end-of-treatment care and how providing such care might benefit both themselves and their patients.

Results indicate two priorities to enable a practice shift: (i) raising awareness of patients’ perspectives and needs around healthy end-of-treatment transitions, and (ii) equipping staff to engage in these conversations. Positive reactions expressed by patients and staff, along with their willingness to use the co-produced webpages, suggest these resources can play a significant role in advancing both goals. Patients and staff viewed the webpages as good awareness-raising tools for staff while also providing practical advice to facilitate care provision. However, due to the complexity and perceived risks of end-of-treatment conversations, bespoke training will likely also be required, and future research can focus on its development and evaluation. Research on training to approach difficult conversations shows the importance of structuring discussions, modelling optimal behaviours, and providing opportunities for practice and feedback (Johnson and Panagioti, 2018).

Although high use of these resources can be expected, they will be unlikely to be sufficient to achieve a normative change in practice. Further investigation is needed to understand how to implement preventive end-of-treatment care as a routine practice in fertility clinics, ensuring it serves a clear function for the different stakeholders (staff and patients). In particular, evidence-based discussions and additional training for staff are necessary to change their current ambivalent attitudes towards the appropriateness of providing this care. Training whereby staff have opportunities to express concerns and acquire resources and skills may be required, or whereby they have opportunities to trial approaches to address concerns (e.g. via role modelling).

While it is likely that patients would benefit from these discussions, not all would be ready and willing to receive them at the early stages of treatment. In line with patients’ expressed preferences and ethical requirements of information provision (Bernat, 2004; Michel and Moss, 2005), it can be argued that preventive end-of-treatment should be offered to all patients at the start of treatment, but staff should explore with their patients if and when they feel ready to receive it, with the reassurance that patients know how to and can easily access such support (Parker *et al.*, 2007; Abdul-Razzak *et al.*, 2014).

### Strengths and limitations

This study addresses an unmet care need using a theoretical, patient-centred, and co-design approach, which proved effective in developing valued resources. Bowen *et al.*’s (2009) feasibility framework enabled a comprehensive assessment of resources’ acceptability and feasibility, while Framework Analysis captured consensual and divergent stakeholder views. The sample size and qualitative analysis ensured data saturation, and the range of positive and negative perceptions reflects a broad understanding of preventive end-of-treatment care. However, most patients were childless, well-educated women recruited via social media support groups, who may have participated because of the value assigned to psychosocial support. Indeed, at the end of the focus groups, most patients acknowledged the benefits of having participated, noting that sharing their experiences had a perceived benefit to their well-being. This aligns with other research, which shows that participants generally value participating in focus groups, often describing the experience as beneficial and even therapeutic (Dyregrov, 2004). Despite efforts to diversify the sample (e.g. Prolific platform and international recruitment), generalizability to men and minoritized groups (ethnicity, socioeconomically disadvantaged, and disabled) is unclear. In these groups, high disparities in access to and acceptability of psychosocial support can be expected due to the lack of financial coverage and resources and high stigma related to infertility and mental health (Culley *et al.*, 2013; Galic *et al.*, 2021). Further development of the present resources to reach a cross-cultural global target population is needed. Furthermore, childless patients are more willing to participate in this research than those with successful treatment outcomes (e.g. Harrison *et al.*, 2021; Sousa-Leite *et al.*, 2023). Further investigation of experiences, preferences, and acceptability testing of end-of-treatment care among this group is important. However, caution is needed in this analysis, as reporting will be retrospective, in contrast with feedback on the experience as it happens (as captured by the present study), and it is expected that (more) favourable evaluations of treatment experiences will be reported (Schmidt *et al.*, 2003). Comparing patients’ views according to their treatment stage (e.g. number of previously completed cycles) was not possible. Still, the results converge with prior research, suggesting limited bias. Staff were representative of the public and private sectors, and their insights were grounded in regular patient contact. Not all suggestions were feasible to be integrated, especially tailoring the resources for minoritized groups, which would require a full assessment of their needs and most likely specific tailored resources (van Balen and Bos, 2004). Future improvements to the resources, including additional features, should be considered.

## Conclusion

There is a high demand for solutions to support the implementation of preventive end-of-treatment care as routine practice in fertility clinics to promote patients’ psychosocial adjustment to unsuccessful treatment. The co-designed educational resources seem feasible and acceptable to address this need in patient care. However, staff expressed ambivalence and concerns about if, how, and when to use them with their patients. Future work should focus on further investigating how best to support staff in this endeavour to ensure such care is provided in a confident, hopeful, and supportive way, so that patients have the opportunity to be fully informed about the realities of their treatment journey.

## Supplementary Material

deaf248_Supplementary_Table_S1

deaf248_Supplementary_Table_S2

deaf248_Supplementary_Table_S3

deaf248_Supplementary_Data_File_S1

deaf248_Supplementary_Data_File_S2

deaf248_Supplementary_Data_File_S3

## Data Availability

The data underlying this article will be shared on reasonable request to the corresponding author.
