# Supplementary material for: A multi-country study to co-design and evaluate digital educational resources to support conversations about ending fertility treatment
Source: Hum Reprod. 2026 Jan 7;41(3):381–93. doi: 10.1093/humrep/deaf248 (PMC13017559; doi:10.1093/humrep/deaf248)
Supplement: deaf248_Supplementary_Table_S1 [file deaf248_supplementary_table_s1.pdf]

Human Reproduction, 2026, 1–1
https://doi.org/10.1093/humrep/deaf248
Supplementary Data

Supplementary Table S1. Composition of each workshop carried out with fertility staff and their sociodemographic and professional characteristics (n = 15).

	**W1 (n = 7)**	**W2 (n = 8)**
**Workshop composition**	7 staffs	8 staffs
**Age** (in years), *M* (*SD*) [range]	44.43 (5.74) [37.00–53.00]	57.50 (15.92) [32.00–75.00]
**Gender identity**	7 women	5 women, 3 men
**Country of residence**	3 Portugal, 1 Belgium, 1 Finland, 1 Italy, 1 UK	2 Brazil, 1 Argentina, 1 Chile, 1 Belgium, 1 Germany, 1 Portugal, 1 Spain
**Professional title**	4 psychologists/counsellors, 2 midwifes/ nurses, 1 clinician	3 clinicians, 2 psychologists/counsellors, 1 embryologist/andrologist, 1 clinic manager, 1 ethicist
**Workplace**	4 public sector, 2 private sector, 1 private and public sector	6 private sector, 1 private and public sector, 1 education/university
**Working in the field** (in years), *M* (*SD*) [range]	14.62 (3.90) [10.00–21.00]	30.55 (13.96) [10.42–49.00]
