# Supplementary material for: A multi-country study to co-design and evaluate digital educational resources to support conversations about ending fertility treatment
Source: Hum Reprod. 2026 Jan 7;41(3):381–93. doi: 10.1093/humrep/deaf248 (PMC13017559; doi:10.1093/humrep/deaf248)
Supplement: deaf248_Supplementary_Table_S2 [file deaf248_supplementary_table_s2.pdf]

OXFORD

human reproduction

*Human Reproduction*, 2026, 1–1
https://doi.org/10.1093/humrep/deaf248
Supplementary Data

**Supplementary Table S2.** Composition of each workshop carried out with patients and patient advocates and their sociodemographic and clinical characteristics (n = 41).

| | W1 (n = 8) | W2 (n = 18) | W3 (n = 5) | W4 (n = 4) | W5 (n = 6) |
|---|---|---|---|---|---|
| **Workshop composition** | 7 patients<br>1 patient advocate | 16 patients<br>2 patients advocate | 4 patients<br>1 patient advocate | 2 patients<br>2 patients advocate | 5 patients<br>1 patient advocate |
| **Age** (in years) *M* (*SD*) [range] | 35.43 (1.90) [32.00–38.00][b] | 39.78 (6.80) [30.00–64.00] | 34.00 (3.32) [30.00–37.00] | 46.50 (10.41) [40.00–62.00] | 39.50 (3.62) [33.00–44.00] |
| **Gender identity** | 8 women | 15 women, 3 men | 5 women | 4 women | 5 women, 1 prefer not to say |
| **Country of residence** | 8 Chile | 18 Argentina | 5 Portugal | 4 United Kingdom | 5 UK[b] |
| **Education** | 8 with higher education | 3 with secondary/ high school<br>15 with higher education | 5 with higher education | 4 with higher education | 6 with higher education |
| **Employment status** | 7 employed, 1 unemployed | 16 employed, 1 unemployed[b] | 5 employed | 4 employed | 5 employed, 1 student |
| **Sexual orientation**[a] | 7 heterosexuals | 10 heterosexuals<br>6 homosexuals | 4 heterosexuals | 1 heterosexual<br>1 homosexual | 4 heterosexuals<br>1 bisexual |
| **Relationship status**[a] | 6 in a relationship<br>1 single | 15 in a relationship<br>1 separated/divorced/widow | 4 in a relationship | 2 in a relationship | 5 in a relationship |
| **Relationship duration**[a] (in years) *M* (*SD*) [range] | 9.65 (5.03) [3.83–16.17] | 9.01 (5.09) [1.00–16.50] | 10.75 (4.91) [3.75–15.17] | 15.08 (2.95) [13.00–17.17] | 8.73 (1.98) [6.50–11.00] |
| **Parenthood status**[a] | 5 childless, 1 with biological children, 1 with stepchildren | 12 childless, 2 with biological children, 2 with stepchildren | 4 childless | 1 with biological children, 1 with stepchildren | 4 childless, 1 with stepchildren |
| **Children from treatment?**[a] | 0 | 1 | – | 1 | – |
| **Fertility treatment stage**[a] | 1 undergoing diagnosis,<br>2 waiting to initiate treatment,<br>2 undergoing (IUI/AI), 2 finished treatment in the past 6 months | 8 waiting to initiate treatment, 6 undergoing (IVF/ICSI), 2 finished treatment in the past 6 months | 1 undergoing (IVF/ICSI), 3 finished treatment in the past 6 months | 1 waiting to initiate,<br>1 undergoing (IVF/ICSI) | 5 finished treatment in the past 6 months |
| **Trying to achieve parenthood**[a] (in years) *M* (*SD*) [range] | 3.61 (2.28) [2.00–8.17] | 3.22 (2.69) [0.83–9.00] | 2.44 (0.39) [2.17–3.00] | 0.71 (0.41) [0.42–1.00] | 5.75 (1.41) [4.25–8.00] |

AI, artificial insemination. IUI, intrauterine insemination. IVF, in vitro fertilization. ICSI, intracytoplasmic sperm injection.
[a] Only for patients (n = 34).
[b] Valid percentages were reported (one participant did not report on their age/country of residence/employment status).
