# Supplementary material for: A multi-country study to co-design and evaluate digital educational resources to support conversations about ending fertility treatment
Source: Hum Reprod. 2026 Jan 7;41(3):381–93. doi: 10.1093/humrep/deaf248 (PMC13017559; doi:10.1093/humrep/deaf248)
Supplement: deaf248_Supplementary_Table_S3 [file deaf248_supplementary_table_s3.pdf]

OXFORD
human reproduction

Human Reproduction, 2026, 1–1
https://doi.org/10.1093/humrep/deaf248
Supplementary Data

Supplementary Table S3. Focus group framework matrix: one row per theme and category, and one column per stakeholders' group (fertility staff; patients and patient advocates).

	Fertility staff (n = 15)	Patients and patient advocates (n = 41)
**Theme: Idiosyncratic, cumulative and protracted burden of fertility treatment can only be addressed with holistic psychosocial care**		
**Category: Fertility treatment pathways are idiosyncratic and unpredictable**	• Staff referred patients' access to treatment depends on the healthcare system patients have, which varies across clinics and countries ('*accessibility [to third-party reproduction] differs greatly, I think, between countries*', W1, N1). Staff also stressed that many '*patients change from one clinic to the other*' (W2, CM1), with or without their advice, which makes it difficult to plan treatment in the long term.	• Patients undergo treatment for different reasons (e.g. health reasons, same-sex couples, single women). The same patient reports different experiences over each cycle, as treatment complications differ and cycles fail in different ways ('*each of them [treatment cycle] failed at a different stage*', W5, Pa1), each new cycle is adjusted according to the patient's clinical history, and different procedures are offered in response ('*I'm going for the second cycle, and this time is through egg and sperm donation*', W1, Pa7). Experiences also vary between patients, depending on the healthcare system they access ('*the prepaid is a struggle because beyond the pain you feel for not being able to be parents, there is a lot of bureaucracy in Argentina with the prepaid*', W2, Pa9), their geographic location ('*the clinic is 230 kilometres away*', W2, Pa8) and financial possibilities (e.g. access to the private sector).<br>• From the patients' perspective, the unpredictability of their treatment journey impacts their psychosocial adjustment during and after treatment and overall satisfaction with the clinic, which for some patients leads them to look for another clinic ('*we're not entirely sure that we will stop, but we have definitely stopped with our current clinic, partly because of how they handled this*', W5, Pa2). Patients referred that clinics should map all possible treatment pathways and share these with patients so they can anticipate and experience the journey more positively.
**Category: fertility treatment has cumulative impacts for all**	Cumulative impacts result from:<br>• Low treatment success rates ('*half of the couples don't get a child from IVF*', W1, CL1; '*three in ten [patients] will not become pregnant*', W1, N1),<br>• Repeated failures and loss, which are extremely difficult for patients: '*I agree that it is very difficult when the result comes back negative, and then negative again, and they keep coming back negative*' (W2, CM1),<br>• Treatment add-ons: '*there are also endless treatments with doing a lot of add-ons which are not successful*' (W2, CL2), particularly in the private sectors, '*because they earn a lot of money with useless add-ons*' (W2, CL2), '*our main problem is the search for money so for treatment*' (W2, EMB1),<br>• Stigma associated with infertility, in particular, in specific cultures ('*I know that for some, in some countries or some subcultures, in western countries as well, there can be a huge stigma on infertility and childlessness*', W2, ETH1),<br>• Impact on patients: treatment greatly impacts patients' emotional well-being ('*patients are very anxious all the time during the treatments*', W2, CM1).<br>• Confrontation with the end of unsuccessful treatment triggers surprise, denial, anger, and frustration: when patients are not offered more cycles, many show '*resistance*' (W1, N1) and anger: '*anger is the number one, number one response*' (W1, CL1), and most patients '*at a certain point, disappear*' (W1, N1).	Cumulative impacts result from:<br>• Low treatment success rates and lack of control over the outcome: '*What I am still trying to learn with infertility that nothing is under our control, nothing*' (W1, Pa7),<br>• Repeated failures and loss, which are extremely difficult for patients: '*the greatest pain I've ever had in my life*' (W1, Pa7), '*grieving on not having the baby but also not becoming a mom*' (W5, Pa4), '*I've had three miscarriages. I can't take it anymore*' (W1, Pa6),<br>• Protracted nature: treatment drags on for a long time due to the long waiting lists ('*about four years' worth of waiting list, and we did not have four years to wait*', W4, Pa1) and because patients undergo multiple treatment cycles and change providers for these cycles ('*move to another clinic for a second opinion, search for a new sort of private consultant*', W4, adv2),<br>• Providers always offer more treatment ('*one more, a little bit more, another donor or double donor*', W2, Pa13), particularly in the private sector, '*because obviously, it is convenient for them as a clinic that you continue because it is still money*' (W1, Pa7),<br>• Complications during treatment and unexpected adverse outcomes: '*My first cycle ended like a surprise ectopic [pregnancy], and I definitely did not feel prepared for the idea that there were other outcomes besides pregnant or not pregnant, and that was a really, really huge shock*' (W5, Pa2),

(continued)

Supplementary Table S3. Continued

	Fertility staff (n = 15)	Patients and patient advocates (n = 41)
		• Impact on patients: fertility treatment is 'an incredibly difficult journey' (W4, adv2), '*physically and mentally brutal*' (W5, Pa1), '*financially and psychologically exhausting*' (W1, Pa1). '*I genuinely thought I was going mental*' (W5, Pa1), particularly after unsuccessful cycles and ultimate unsuccessful treatment. Physically, patients feel they have '*lost control of your body (…) no autonomy over it*' (W5, Pa1),<br>• Lack of formal and informal support: patients feel '*the process is super lonely*' (W1, Pa3), even more so during the Covid-19 pandemic ('*my husband couldn't go in, so I received the news alone. It was very hard. I left heartbroken from there*', W1, Pa4), and in particular after unsuccessful treatment. A minority of patients share the process with their close ones, but overall, patients feel unsupported and do not feel understood, even by the staff. Patients feel '*retreated into like an IVF bubble (…) I don't feel there was anybody I could talk to about it because nobody understood what it was like to stab yourself every day*' (W5, Pa1),<br>• Impact on the partnership: '*how my husband supported me, and, in some sense, he didn't know how or wasn't prepared or didn't on some level*' (W5, Pa1),<br>• Confrontation with the end of unsuccessful treatment triggers surprise, denial, anger, and frustration: patients go through an unexpected roller coaster of emotions: '*I never expected to respond the way I did [when treatment was unsuccessful] (…) my world fell through the floor when I got the news, and I've never known a feeling like it, and it is the most isolating thing in the world even if you were doing it as a couple*' (W5, Pa1). Patients feel they '*completely lose my [their] identity*' (W5, Pa1) and go through an '*existential crisis*' (W5, Pa2). They feel '*really mad that I wasted the last like four years of my life (…) I didn't look after me, didn't do the things I should have and didn't have joy in my life*' (W5, Pa2). Patients need some time to acknowledge this event and '*reconnect with yourself and your life and prioritise you and self-care*' (W5, Pa1). They felt the frustration associated with unsuccessful treatment was shared by everyone involved, '*the consultant, the clinic, everyone who wishes results are always positive*' (W3, Pa2).
**Category: satisfying care experiences require empathic and holistic psychosocial care, including preventive end-of-treatment care**	Staff referred that empathic and holistic psychosocial care that covers the whole treatment pathway and includes preventive end-of-treatment care is associated with a higher quality of patient-centred care and patients' overall satisfaction with the care provided.<br>**Empathic and centred care**<br><u>Positive determinants</u>:<br>• <u>Empathic and timely support during and in the immediate aftermath of the end of treatment</u><br>In some private clinics, '*the medical colleagues always call the patients two, three days after the procedure to check if everything is fine*' (W2, CM1), and the mental-health team contacts patients and '*try to we try to make us [psych team] always available for patients every time is possible*' (W2, Psych2).<br>Staff referred preventive end-of-treatment care should be offered in an empathic and sensitive way ('*It has to have a great professional, empathic way. So, yes, I think maybe it's not as simple as that*', W2, Psych2). It should be delivered according to the patient's level of differentiation '*by talking with them or writing [information] for them*' (W2, CL3), and the language used also need to be sensitive throughout to avoid blame or put extra distress on	Positive and negative experiences of empathic and holistic psychosocial care that covers the whole treatment pathway and includes preventive end-of-treatment care are described below, which, in patients' views, are associated with better quality of and higher satisfaction with the clinic-provided care:<br>**Empathic and centred care**<br><u>Positive determinants</u>:<br>• <u>Supportive, empathic, and responsive relationship with the staff</u><br>Feeling validated and supported by staff ('*the doctor was very warm, very empathetic*', W2, Pa4; '*very kind, after a negative result they [the clinician] always asks me how I am psychologically*', W3, Pa3),<br>Having someone to turn to at any time during and after treatment ('*the midwife has been like my support, let's say. I chat with them [the midwife] on WhatsApp. And I think they have been my fundamental support throughout the treatment*', W1, Pa1).<br>Patients referred that <u>preventive end-of-treatment care</u> should be carried by respectful, sensitive, and trustworthy staff members and with time ('*needs to be considered, it needs to be respectful, and it needs to*

(continued)

Supplementary Table S3. Continued

	Fertility staff (n = 15)	Patients and patient advocates (n = 41)
	patients (‘*like dropout rates, for example, it already suggests that you are kind of giving up and that you’re not doing enough to fulfil your goal. Whereas it might be that some patients stop because it’s very emotionally draining, and they have other things in life that they want to focus on now. And so maybe if we can also try to see, you know, portray this more positively, that you can have a positive outcome without children, that might help the patient*’, W2, ETH1).<br>Negative determinants:<br>• Lack of emotional support provision after unsuccessful treatment: ‘*After they [patients] finish [treatment], they feel abandoned, maybe the clinic make a phone call to tell them [patients] that treatment was unsuccessful, but does not offer, for example, further discussion*’ (W1, Psych1).<br>**Organization of care (personalization)**<br>Positive determinants:<br>• Access to holistic care<br>Staff working in the private sector tended to describe and value more holistic, multidisciplinary, and personalized practices. Patients tend to have contact with all staff members, ‘*we also have psychologists. When the patient starts ovarian stimulation, we create a WhatsApp group. We have a clinician, we have people from orientation [counselling], there is a pharmacologist and a nurse [in the WhatsApp group]*’, (W2, CL3).<br>Negative determinants:<br>• Lack of access and screening/referral to medical and mental health care<br>Usually, patients are not offered support (both medical and psychosocial), in particular after unsuccessful treatment, ‘*it is the patient who has to ask for an appointment*’ (W1, Psych1). Staff only tend to refer those patients who show significant distress for support (‘*If we’re trying to convince them [patients] to stop and they’re resistant, then we might offer them a consult with the psychologists of our centre*’, W1, N1).	*be given the time that it deserves*’, W4, adv2).<br>Patients would like preventive end-of-treatment care to be offered in particular in an individual/couple format, respecting patient’s values, needs and preferences (‘*person-centred tailored to the individuals, so they [staff] can make a clinical judgement about the person, the people sitting in front of them, their situation and shape it*’, W5, Pa3).<br>• Receiving emotional support<br>Receiving emotional support (‘*they [the clinic] do have psychology sessions included in the treatment*’, W1, Pa7) and, in particular, group support (‘*It was a mutual support group with different couples with a series of experiences that have truly been one of the best things we have had in this process, especially before trying any treatment*’, W2, Pa3) was considered by all patients essential and highly beneficial. Those who received it (most of whom were outside the clinic) considered it helpful in making them feel validated and understood and in becoming aware of and coping with the rollercoaster of emotions triggered by treatment.<br>Negative determinants:<br>• Lack of empathy of staff<br>No validation of emotions: ‘*there’s no containment for how hard it is*’ (W1, Pa6). ‘*Inappropriate*’ (W1, Pa6) comments and lack of ‘*empathy*’ (W1, Pa2) from staff when sharing adverse outcomes or talking about treatment procedures or available future options, ‘*they [staff] remain very cold*’ (W1, Pa3), ‘*there is no human warmth there, no attention because sometimes a word is enough, isn’t it!?*’ (W3, adv1), ‘*it was like an administrative process*’ (W2, Pa7),<br>High workload ‘*sometimes it is one more patient on a list of 20*’ (W3, adv1). Patients felt they were treated as in a ‘*conveyor belt*’ (W5, Pa1, Pa5). The care was provided in a rush, ‘*which is not acceptable at all in these circumstances*’ (W4, adv2), ‘*losing that personalised touch*’ (W5, Pa5).<br>Communication of the negative treatment outcome. Some patients find out about the treatment outcome by phone, others by email or letter, and others find out for themselves and still have to communicate the result to the clinic. Patients considered these methods not ideal, ‘*leaving patients very, very isolated*’ (W3, adv1).<br>Regardless of how treatment results are communicated (in-person, by phone, email, being the patient who communicates it to the clinic), patients do not feel supported nor validated, ‘*I started crying during the phone call [to inform about an unsuccessful cycle], and there wasn’t even nearly a validation of my feelings*’ (W3, Pa1).<br>• Lack of emotional support provision<br>Most patients feel ‘*support was totally deficient*’ (W2, Pa1). Many patients referred they ‘*have not had any support from the clinic of any kind*’ (W1, Pa5), especially poor after unsuccessful cycles and treatment: ‘*they [clinic] only called me to say that it failed. There is no session to talk about it*’ (W1, Pa2), ‘*the support really was zero*’ (W2, Pa5), ‘*they just left us*’ (W5, Pa1).<br>**Organization of care (personalization)**<br>Positive determinants<br>• Access to holistic care<br>Having a multidisciplinary team: ‘*the doctor, the psychologist, and the endocrinologist*’ (W2, Pa4),<br>Patients referred that preventive end-of-treatment care should be ‘*approached in a much more holistic way, seeing the patient as a whole and with a multidisciplinary team*’ (W3, Pa2),

(continued)

Supplementary Table S3. Continued

	Fertility staff (n = 15)	Patients and patient advocates (n = 41)
		Overall, private clinics were perceived as more supportive (more resources and personalized care), but even so, they were insufficient.<br>Negative determinants<br>• Lack of coordination and continuity of care<br>Patients felt staff '*don't work as a team*' (W2, Pa3). Most patients '*had contact only with the doctor*' (W1, Pa5) or felt '*there was no articulation between psychologists and clinicians*' (W1, Pa2). The care '*was very medically oriented, professionals [clinicians] didn't have training on the psychological impact of treatment*' (W5, Pa4). There was also no continuity of care ('*I also changed doctors constantly*', W1, Pa6; '*I had to re-explain stuff, and then it's all getting lost in translation*', W5, Pa5).<br>• Lack of access and screening/referral to medical and mental health care<br>Long waiting lists for mental health support ('*six-month waiting list? I need someone to talk to now*', w4, Pa1), low frequency of support, and deficient referral processes ('*they [the embryologist] did say that they would refer me to the counsellor, but then I never got any contact*', W5, Pa1). Patients highly endorsed this latter constraint. Most patients referred that it is the patient '*who has to insist on*' (W1, Pa1) support (both medical and mental health), in particular after treatment. Some patients '*feel like I'm begging. I feel like I'm wasting their time*' (W1, Pa2). Many patients ended up self-referring outside their fertility clinics ('*the psychological help that I have taken has been because I feel that I can't take it anymore, but not because my doctor has told me to go to a psychologist*', W1, Pa6) but acknowledged there are high financial costs associated with it.
**Category: patients who feel empowered to take control over their treatment and parenthood decision-making have more satisfying care experiences**	Staff also referred that different perceptions about shared decision-making contribute to higher quality of patient-centred care and patients' overall satisfaction. Positive and negative determinants of care are described below:<br>**Shared decision-making**<br>Positive determinants:<br>• Timely information provision<br>In some private clinics, staff give patients opportunities to discuss their concerns during and after treatment ('*We always ask them to come back [after unsuccessful treatment] to talk to the doctor*', W2, CM1).<br>Negative determinants<br>• Lack of information provision<br>After unsuccessful treatment, patients do not have an opportunity to discuss their treatment and future plans, '*clinics make a phone call to tell them that treatment was unsuccessful, but do not offer, for example, further discussion (…) an appointment to discuss what happened at the end (…) They [patients] need a sort of follow up meeting with the clinic, with the doctors to understand and close the process. I notice this gap in care*' (W1, Psych1),<br>Patients need to seek information on their own ('*I don't know how it is in the UK, but here in Finland, people devour all the information available. Any information and all the information they [patients] need: Finnish sources, English language sources, any sort of sources*', W1, CL1).	Patients described different experiences of shared decision-making, which contributed to more positive perceptions of the quality of care and higher satisfaction with it. Positive and negative experiences are described below:<br>**Shared decision-making**<br>Positive determinants:<br>• Information provision and shared decision-making<br>Patients valued having the opportunity (during and after unsuccessful treatment) to be encouraged and given the time to discuss their concerns and decide about their treatment and future options. Patients felt their consultant '*answered all our questions*' (W2, Pa8) and '*gave us a range of possibilities*' (W2, Pa9). Patients referred that '*although the result itself was the same [negative] (…) the way we were treated was what made the difference*' (W2, Pa14).<br>Negative determinants:<br>• Lack of opportunities for shared decision-making<br>Patients did not feel involved in their treatment plan ('*I need to feel much more listened to*', W1, Pa2) and did not feel they had the information needed to make informed and timely decisions ('*But what is that? I mean, why don't they [clinics] talk to me about it before I've made my decision?*', W1, Pa4), impacting their overall satisfaction with and evaluation of the quality of the provided care. Although treatment-informed consent presented information on probabilities and risks of treatment, patients felt it did not promote autonomy: the consent is associated with the '*legal part of treatment*' (W3, Pa1), and the information is not presented nor discussed in a comprehensive way ('*Although they [clinic] tell you, for example, you can ask questions and anything, you can ask the midwife or your doctor before signing [the informed consent], I feel that it is like they*

(continued)

Supplementary Table S3. Continued

	Fertility staff (n = 15)	Patients and patient advocates (n = 41)
		give you the papers and it's like read it and sign it and hand it in', W1, Pa4),<br>Patients felt they '*don't have the tools to decide*' (W2, Pa2); '*we are not entirely sure [when] we will stop*' (W5, Pa2). Patients '*are thirsty for answers and information*' (W3, Pa2) but receive '*little information from the beginning*' (W1, Pa4). Patients felt staff '*skimp on information*' (W2, Pa2), with some perceiving '*they [staff] assume we already know and don't tell us or assume we don't care*' (W3, Pa2). The information was overall insufficient (not covering treatment procedures, reasons for unsuccessful cycles/treatment, continuing vs discontinuing treatment, alternative options and pros and cons), given about the next treatment step and not tailored to the patient's circumstances ('*not knowing and not getting answers it's difficult throughout the journey, but (…) near the end it gets more difficult and more painful and very much more heightened*', W4, adv2; '*they didn't give us any warning about what it would be like before the failed cycles, just as Pa1 said. We went into our third cycle, saying: —"This is it, this is our last one. We want to pull out all the stops, you know, leave no stone unturned, tell us everything" (…) and in the follow-up chat, they were like: —"Oh well, here's this other thing that you could do," and we were like: —"Why didn't you tell us this before? We were very serious that this was our last time, we have no more money." Then they immediately suggested donation with like no warning*', W5, Pa2).<br>• <u>Sub-optimal information delivery/communication</u><br>When information was shared, it was many times overwhelming, as it was provided at once ('*she was always talking really fast, and I was trying to take in this information, it was just hard, it was like information overload*', W5, Pa5; '*many words that you don't know*', W1, Pa2), with no preparation and support ('*I think our heads weren't ready to process it. I think that's where there was a lack of support*', W2, Pa13), Patients perceived staff were not at ease in sharing the negative treatment results, '*you can see their discomfort in giving you a negative*' (W2, Pa3). Patients referred they lack information on the possible negative complications and treatment outcomes. Most treatment-related discussions focused on '*what the next [treatment] step will be*' (W1, Pa5), with little consideration of the overall treatment trajectory<br>Therefore, patients felt they had to do their '*own research*' (W4, Pa1) (using webpages, social media forums, webinars, TED talks, books) '*to search a little for those answers that you can't find*' (W1, Pa3) '*about treatments, about embryology, about a bunch of things*' (W2, Pa2), but were unsure if they were accessing a reliable source and all the required information. '*no one explains*' (W3, Pa4) about intermediate cycle complications (e.g. failed stimulation, oocyte pickup, fertilization), '*each of them [treatment cycle] failed at a different stage, and that completely caught me off guard, which kind of enhanced worsened that kind of trauma response really*' (W5, Pa1).
**Theme: Preventive end-of-treatment care is important, but its risks and benefits are not consensual**		
**Category: Conversations about the possibility of unsuccessful treatment are not the norm**	Staff referred that '*we often don't have that conversation*' (W1, N1) about the possibility of unsuccessful treatment:<br>• Staff only discuss this possibility with '*very, very, very few patients*' (W1, CL1), only with those who are '*certain they will not have more treatment, with you nor anywhere else*' (W1, N1),	• The possibility of cycle and unsuccessful treatment is rarely discussed. Staff only tend to briefly acknowledge '*only the treatment success chances (…) but always talking about it as if it was definitely going to be successful*' (W4, Pa2) ('*I remember asking the doctor in one of the appointments what would happen, so what the next [treatment] step would be if it [the treatment cycle] didn't work, and he even said to me: —oh,*

(continued)

Supplementary Table S3. Continued

	Fertility staff (n = 15)	Patients and patient advocates (n = 41)
	• Some staff compared this conversation with conversations about ‘stopping treatment’, which ‘*in these 22 years working in the field, I almost never heard, from the fertility clinic side or the patients’ side that they want to stop treatment, or from the clinic side, suggesting patients to stop treatment*’ (W2, Psych1),<br>• Notwithstanding, staff highlighted they mention this possibility, particularly with patients with very poor prognoses. The standard information was about the treatment success rates (formulated in terms of the probability of treatment working and not of not working). However, staff perceive patients do not acknowledge this information ‘*it’s like we’re talking to a wall*’ (W2, CM1), as ‘*couples don’t internalise these, they always think: —okay, it may not work, but it’s going to work for me*’ (W1, Psych4),<br>• Only one psychologist referred they discuss the possibility of unsuccessful treatment with those patients who ‘*come to me saying they want to work on a kind of coping strategies and planning for if treatment doesn’t work*’ (W1, Psych2),<br>• Reluctance to label cycles as the last attempt: staff referred that ‘*the clinics are reluctant to tell it’s the last one [cycle]*’ (W1, Psych4), as it is difficult to know when treatment will end (‘*the difficulties are reaching the end of the road, rather than being at the end of the road*’, W2, CL1). This is mainly due to the idiosyncrasy of treatment (‘*no one says that it’ll be the last IVF treatment, we [staff] always offer other options like embryo donation*’, W1, N2). Staff perceive patients ‘*don’t like to stop and the physicians as well*’ (W2, CL2).<br>• Staff referred that patients need to be given hope to be able to pursue treatment and not focus on the negative side of treatment. Some referred to that ‘*because infertility is often compared to cancer in terms of the burden. I think what we currently do is follow more or less the same strategy, which is to offer to always concentrate on what can be done and not really on what cannot be done*’ (W1, CL2).	*let’s not think about it now, like, let’s be optimistic because it will work (…), so in my case, it is unexplained infertility, so the doctor was quite optimistic and did not even want to address the scenario of not being successful*’ W3, Pa1). ‘*Preparation for failure was completely inexistent*’ (W3, Pa1) at the start and during the whole treatment pathway (‘*I don’t recall any preparatory conversation for it if it fails or what that might be like*’, W5, Pa1; ‘*Never in my ever, in my experience, and I did see loads of doctors, I can tell you that*’, W4, Pa1),<br>• Gametes/embryos donation is only discussed at the end of treatment, and as the last resource (‘*then they immediately suggested donation with like no warning*’, W5, Pa2), ‘*other possibilities*’ (W1, Pa6), such as adoption or child-free lifestyle, were rarely discussed (‘*It is a personal issue that surely not everyone wants, but like it isn’t mentioned either*’, W1, Pa6), and the possibility of ‘*stopping trying is never mentioned*’ (W2, Pa13),<br>• Three patients reported they discussed the possibility of unsuccessful treatment. They discussed it with their psychologist, considering it challenging but very helpful (‘*with the psychologist, we have worked a lot that we really realise that there is a life without children, that you can also be happy, that you can do a bunch of things*’, W2, Pa10).
**Category: such conversations are needed and beneficial, but staff and patients often have differing views on the risks and the extent of the benefits**	Staff recognized the importance of discussing the possibility of unsuccessful treatment with their patients (‘*It is really important that we do mention it because we know that at the end of a treatment pathway, even if they [patients] do six cycles with us with us in Belgium, three in ten will not become pregnant*’, W1, N1), but expressed some ambivalence and concerns about it:<br>• They also considered such discussions could be seen as inappropriate (particularly at early stages of treatment) because patients are too invested in treatment (‘*they’re still wanting to have treatments or still considering*’, W1, N1),<br>• Staff expressed additional concerns about the possibility of these conversations triggering negative emotions in patients (‘*if you concentrate on what cannot be done with treatment (…) when you stop the treatment, then I can see how the couple disintegrates because they want to explore other possibilities, other clinics abroad or anywhere else (…)*’, W1, CL1) and reducing their satisfaction with the clinic (‘*they would be extremely angry*’, W1, CL1),<br>• One psychologist stressed that ‘*until you’re actually there [patients are confronted with unsuccessful treatment], I just think it’s actually quite a difficult question or a difficult thing to plan for*’ (W1, Psych2),<br>• Although staff recognized patients would benefit from being aware of the possibility of unsuccessful treatment from the start, actual preparation and planning for this possibility were considered	Patients ‘*do feel a great need to prepare for the possibility that nothing works or that each [cycle] won’t work*’ (W2, Pa11), considering ‘*without a doubt*’ (W3, Pa1) that these conversations would be helpful:<br>• Although patients recognized these ‘*conversations are hard*’ (W4, adv2) and discussing unsuccessful treatment would be difficult for both patients and staff, as it could trigger negative emotions, patients did not consider this potential downside should prevent conversations from happening, as ‘*not having any conversation at all about the impact of it failing would lead to bigger trauma then, if experienced*’ (W5, Pa1),<br>• Patients ‘*can’t think of any situation where they [staff] shouldn’t [have these conversations]*’ (W5, Pa3). However, they acknowledged that the moment and how it is delivered is important and can impact patients’ reactions towards it (‘*I think it’s very useful but (…) sometimes, there are several phases of the treatment that I sometimes prefer not to know, because my anxiety interferes a lot with the process*’, W3, Pa2),<br>• However, although all patients would like to be prepared for the possibility of unsuccessful treatment, a minority of patients also recognized it is difficult to prepare patients in advance and wondered if there is any ‘*amount of talking about it before that could really prepare you*’ (W5, Pa2).<br>Some patients also acknowledged that patients’ willingness to receive preventive end-of-treatment

(continued)

Supplementary Table S3. Continued

	Fertility staff (n = 15)	Patients and patient advocates (n = 41)
	difficult while patients were pursuing treatment and would be more appropriate at later stages of treatment ('*I think it would help in the beginning just to have a first conversation of the options that we have here and be available to discuss them along the way of the treatment*', W2, Psych2). However, staff also agreed that only providing this support after unsuccessful treatment would not be optimal as '*what we see is that the patient, at a certain point, disappears*' (W1, N1).	care may be a '*very individual thing*' (W4, Pa2) and might '*there are people who won't be so comfortable talking about it and others who will be*' (W3, Pa1). However, it needs to have a '*delicate balance*' (W5, Pa2) between realism and hope, as patients need '*the energy and the hope, you know, to get through an incredibly difficult journey*' (W4, adv2).
**Category: staff lack resources and know-how to engage in these conversations**	Lack of resources:<br>• Staff do not feel confident in discussing the possibility of unsuccessful treatment with their patients because they do not have the required resources to do so ('*they [patients] can be offered, of course, a visit to the psychologist, like in Belgium, but I think nothing more than this*', W1, CL1),<br>• Staff stressed that '*the reason why many clinics don't have that conversation early enough is because they don't have anything to really offer*' (W1, N1).<br>Lack of training/communication skills:<br>• Staff considered discussing the possibility of unsuccessful treatment '*a difficult job*' (W1, CL1) and stressed their lack of training in doing so ('*the majority of the doctors are not trained right from the beginning to approach this topic with their patients*', W2, CL2).<br>• Most staff find it challenging to know how much information they should deliver about the possibility of unsuccessful treatment, how to provide this information, and to what degree they should tailor its delivery to the patient's profile. Staff also stressed the lack of evidence about the right time to discuss the possibility of unsuccessful treatment ('*we do not know. And I think that is one of the main difficulties of this: we have no studies addressing exactly this question and with good controls, with enough psychological support before treatment*', W2, CL1).	Lack of resources:<br>• Patients highlighted they do not have access to information about the possibility of unsuccessful treatment (nor in the clinic, nor online) ('*there is so much in the whole world. But this, the aftermath [of unsuccessful treatment], there is nothing. If you search for it, there's nothing*', W2, adv1),<br>• They also mentioned clinics lack resources to signpost patients for support for unsuccessful treatment: '*the clinic was also not prepared [for unsuccessful failures/treatment]. Like they had no information for me, they really didn't know how to support me (…) They really didn't have like any road map to explain this to me*' (W5, Pa2).<br>Lack of training/communication skills:<br>• Patients highlighted staff's '*lack of training*' (W5, Pa4) and communication skills when addressing and providing support for the emotional burden of unsuccessful cycles and ultimate unsuccessful treatment. Patients recognized '*the medical nature of it [treatment] is where people [consultants] feel comfortable and are trained in*' (W5, adv1) but stressed the medical team should also '*take responsibility as well as take charge*' (W1, Pa1) for the emotional care of treatment.<br>Patients indicated that staff lack awareness of '*patients' perceptions, what they feel because sometimes they [staff] might know, but this needs to be more explained, they need to put themselves in the other's [patients'] place, other's shoes, and maybe reinforce this message that it is important to have this approach*' (W3, Pa2).
**Theme: The function of preventive end-of-treatment care**		
**Category: ensure patients feel prepared and supported in moving through the grief and cope with short-term associated challenges**	• Staff recognized it would be helpful to prepare patients for the possibility of unsuccessful treatment because '*even though it's difficult in the beginning because you want to give them hope, it might be easier for them at the end of the trajectory*' (W2, ETH1).<br>Via information on (un)success rates:<br>• Some staff highlighted the need to explicitly inform about the likelihood of treatment not working and not only the likelihood of working ('*when we tell a patient that she has a 30% chance of becoming pregnant or delivery, she, we are not addressing that she has 70% chance of not becoming pregnant. And that is a very different thing. And people hear what they want to hear*', W2, CL1). Some stressed it is important to provide information about both single cycle and cumulative success rates, and specific to the type of treatment (with or without donation),<br>• Two psychologists referred that patients should also discuss the possibility of continuing vs discontinuing treatment ('*it is a very difficult decision, and some couples and some people need just some support here*', W2, Psych2).<br>Via support sources:<br>• Staff considered preventive end-of-treatment care would be an opportunity at the '*very beginning of the treatment trajectory*' to signpost patients for	• It was consensual among patients that receiving preventive end-of-treatment care at the start would help them better cope with the treatment journey and unsuccessful treatment if it comes to happen ('*I think accompaniment or support should be considered from the beginning, so that you can have the tools to face those times when the treatment fails*', W1, Pa1),<br>• Patients felt that being prepared for unsuccessful treatment would be beneficial to '*have more knowledge*' (W1, Pa5), '*not being given false expectations*' (W2, Pa2), '*receive more psychological support*' (W1, Pa5), '*make more informed decisions*' (W5, Pa1), and have the '*confidence*' (W1, Pa1, Pa5) and '*the tools to face those times when treatment fails*' (W1, Pa1),<br>• Patients also stressed that having these conversations would positively impact their satisfaction and trust in the clinic: '*it would also give me confidence that even if something went wrong, they would be right there*' (W3, Pa4).<br>Via information on individual prognosis, all treatment options and complications:<br>• Be informed about individual treatment success rates and prognosis,<br>• Be informed about '*how many rounds of treatment*' (W4, adv2), '*all [treatment] options*' (W2, Pa11), and '*what [treatment] options do we have if treatment is unsuccessful*' (W3, Pa1),

(continued)

Supplementary Table S3. Continued

	Fertility staff (n = 15)	Patients and patient advocates (n = 41)
	'some support to go on with their life' (W1, N1) in case of unsuccessful treatment ('*If you're among those 30 unlucky per cent, then we'll also, you know, offer you some support to go on with your life. I think that would be like a really important thing to offer, and it could even help us (…), because we might be brave enough to say that to the patient because we can offer them some support afterwards… because it's all interconnected*', W1, N1).	• Be informed about the treatment procedures and all possible complications: '*good explanation of all steps of treatment*' (W3, Pa4), '*all scenarios*' (W3, adv1) and '*what can go wrong at each step*' (W2, Pa2) because '*what we could control is to be clearly informed about what can happen from medicine*' (W1, Pa7),<br>Via holistic support to cope with the emotional, relational, and social impact of ending treatment:<br>• Be informed about the emotional burden of unsuccessful treatment ('*get to know those feelings that happen to you*', W2, Pa4),<br>• Be informed about coping strategies to manage the emotional burden of unsuccessful treatment ('*what you could do in those circumstances to cope with feelings of grief*', W5, adv1),<br>• Be informed about how to manage social relationships—insensitive comments, questions, others with children or reaching parenthood ('*how my relationship will change with other people because when I was trying, loads of my friends were having like successful pregnancies*', W4, Pa1),<br>• Be informed about how to manage challenges in the partnership—communication and mutual support ('*difficulties in how my husband supported me (…), what this means for you as a couple, and what your motivations are so when it fails, they can like lay on these*', W5, Pa1),<br>• Be reassured and signposted in advance to different types of support because '*for some [patients] it may be just having other types of activities, others will benefit from a support group, others from psychological support*' (W3, Pa1). Psychosocial support was particularly mentioned: '*it should be considered from the first moment you begin treatment*' (W1, Pa1) and '*even in the private sector, psychological support should be included in treatment*' (W3, Pa3). Group support was also very much emphasized, as '*it could be a plus if the clinic could put together a group of people who are in the same circumstances where there are options to talk, to meet, basically like what each one feels and share experiences*' (W1, Pa3).
**Category: ensure informed consent**	• Staff considered patients would not be prepared to discuss this possibility at the start, as this is a moment when there are high chances of success, in which patients have a lot of hope and are very engaged: '*because I oftentimes think in the beginning, patients are given a lot of hope. You don't want to say: well, you know, it'll probably not work, but we'll try to treat you. So, you'll say—We'll do everything we can to help you*' (W2, ETH1). They referred that preventive end-of-treatment care at the treatment start is part of the informed consent in the way that '*it's all part of explaining success rates of treatments*' (W1, N1).<br>• Staff were concerned about how in-depth the possibility of unsuccessful treatment should be addressed, referring to the fact that it should not superimpose the hope for successful treatment ('*we need to have this fertility hope, feel that we are moving on every single day*', W2, Psych1),<br>Follow-up provision:<br>• Notwithstanding, some staff considered that having a follow-up meeting with the medical team to review the process and be informed about the reasons why the cycle did not work is needed ('*patients need a sort of follow-up meeting with the doctors to understand and to close the process*', W1, Psych1).	At the start:<br>• Patients considered discussing the possibility of unsuccessful treatment and its psychosocial implications '*vitally important at the beginning before treatment commences*' (W4, adv2). Patients indicated '*are part of the informed consent*' (W2, Pa1), part of the '*decision tree*' (W3, Pa1). '*It's my right to be informed from the beginning, perhaps that I am going to undergo treatment and how these things can happen, and then I give consent that I want to undergo this treatment assuming those risks and knowing*' (W2, adv2),<br>• These conversations about unsuccessful treatment need to be very informative, '*open and honest*' (W4, adv2) to enable patients to be '*in charge of their own health, the treatments, and the choices*' (W2, Pa2).<br>With follow-up provision:<br>• Notwithstanding, having a follow-up meeting with the medical team to review the process and be informed about the reasons why the cycle did not work. Patients would like to have a '*structured closing of the cycle*' (W1, Pa7), '*here's everything we can tell you (…) it would be important for the clinics to offer closure of what happened, like an analysis, a reflection*' (W1, Pa2). For some patients, knowing there is no explanation is important to close the process ('*you know what, there isn't an answer. But for me, that is an answer*', W1, Pa7).

(continued)

Supplementary Table S3. Continued

	Fertility staff (n = 15)	Patients and patient advocates (n = 41)
		Depth should be tailored to the patient's needs and preferences:<br>• Overall, patients expressed that having this awareness from the start would be essential, and some stressed they would benefit from being given the opportunity to revisit it as they progress through treatment cycles. During the actual treatment process, some patients said they would not '*welcome too many of those conversations*' (W4, adv2). In addition, although all patients would like to receive preventive end-of-treatment care and agreed it should be offered to all patients at the early stages of treatment, some also stressed that some patients might only be prepared to have more in-depth conversations at later stages.
**Category: explore other pathways to and beyond parenthood and re-orient one's life goals**	• Two staff said that patients should be informed about alternative (parenthood) pathways beyond treatment and a child-free lifestyle ('*if it doesn't work, there are really good options for you. There are other options of having a child or having a life, you know, without children that can also be fulfilling*', W2, ETH1)<br>'*Yes, I think for most people, it would help to have a whole view at the beginning (…) of the trajectories that they can go on (…) a first conversation of the options that we have here and be available to discuss them along the way of the treatment*' (W2, Psych2). From their perspective, having alternative fulfilling life goals not only can support people if they face an unsuccessful treatment but also help them decide to continue vs end treatment. '*the question is, what is at the end of the wish?*' (W2, CL2). '*If people manage to get new life goals ahead of them that they can focus on rather than having a child, they can also have a happy and fulfilling life. And I always wonder if we take that into account enough at the beginning of the trajectory*' (W1, ETH1).<br>• One staff additionally contested references to unsuccessful treatment, questioning what successful means and referring to that ending treatment (even without achieving a live birth) '*sometimes is a success!*' (W2, CL2), as patients reached acceptance of their unfulfilled desire for children or found alternative ways to fulfil this wish ('*Very often, I feel happy that we can stop it*', W2, CL2)	• Be informed about alternative (parenthood) pathways '*so, what's plan B? If plan A doesn't work, what will be plan B, or plan C or plan D?*' (W3, adv1), '*other ways of being able to be a mother*' (W2, Pa4), '*other paths for people who do think about it [other paths beyond treatment to achieve parenthood, such as adoption] and who maybe need like that little push or that encouragement*' (W1, Pa6).<br>• Be informed about a child-free lifestyle ('*I may not have children in my life, so what, why, and how can I bring meaning?*', W5, Pa4).
**Category: signposting patients for psychosocial support**	• Staff envision preventive end-of-treatment care as a means to signpost patients for support. They referred this should be provided by the medical team with a signposting for mental healthcare professionals because this latter would have more expertise and training in exploring the emotional issues around the possibility of unsuccessful treatment and provide support for patients during the whole treatment process and in the aftermath ('*I think, having been doing this job for a lot of years, the main problem from my point of view is to differentiate between those things that the doctor should do and those things that a psychologist should do*', W2, CL2).	• Patients considered preventive end-of-treatment should be provided by the medical team (all staff with an active role in treatment), the mental health team, and promoted by the clinic as an institution (e.g. actively offered by the clinic): '*holding a team meeting, let's say because here is not only the gynaecologist but there's also the psychologist, there's the psychiatrist in some cases, there's the urologist as well. So, there are many doctors and those who participate in medical health who have an important role*' (W1, Pa5). However, patients acknowledged that '*obviously, I don't expect a consultant to be a psychologist*' (W3, Pa1) and stressed that mental healthcare professionals would be more trained to support patients exploring some topics on the possibility of unsuccessful treatment.
**Theme: Co-designed digital educational resources can support the routine provision of preventive end-of-treatment care at clinics**		
**Category: digital educational resources support the provision of preventive end-of-treatment care**	• All staff considered most patients, '*not all of them of course*' (W1, CL2), '*would definitely want to explore that in their own surroundings and time*' (W1, Psych2).<br>Staff perceived the digital educational resources were highly useful and helpful for both patients and themselves and identified several benefits from them:	• All patients were willing to explore the educational resources ('*from what I know, I think that it will be really well received*', W4, avd1). Patients valued that the resources were self-guided and that patients could explore and reflect on them on their own and at their own pace (before or after having discussed with their medical team; '*without a doubt that [after the clinical appointment] I would be*

(continued)

Supplementary Table S3. Continued

	**Fertility staff (n = 15)**	**Patients and patient advocates (n = 41)**
	• Offering a gentle approach to the possibility of unsuccessful treatment and, therefore, promoting preventive end-of-treatment care provision at fertility clinics,<br>• Providing information to support staff delivering preventive end-of-treatment care. Staff highlighted it would be '*helpful not only for clinicians but for all clinic staff*' (W2, CL3), '*for training purposes for staff (…) to reflect on those questions [common questions and concerns section] which could be addressed to the patients*' (W2, CL2),<br>• Benefiting patients: '*it would be really good for the patients*' (W2, ETH1). It would help patients find psychosocial support and validate their emotions and experiences by '*sharing and speaking about these [these emotions and experiences]*' (W1, Psych1).<br><br><u>Perceived barriers do not outweigh benefits</u>:<br>• Staff did not consider any potential adverse effects of the digital educational resources for themselves but stressed they could '*raise some anxiety*' (W2, Psych2) in some patients.<br>• Staff were willing to offer them to their patients but expressed concerns about exploring them in the consultation due to lack of time, appropriateness, and training. In particular, staff were concerned about how and to which patients the resources should be offered: '*how can this project fit in reality? When clinicians are with a patient in front of them, how would they share this information?*' (W2, Psych1),	*curious to explore these better at home, in a private and safe place*', W3, Pa1),<br><br><u>Patients identified several benefits from the digital educational resources</u>:<br>• Promoting preventive end-of-treatment care provision at fertility clinics: '*clearly helps to open or start the conversation with the clinic*' (W5, Pa4),<br>• Providing the required information to support staff in preventive end-of-treatment care provision ('*it provides key points to be considering when you're talking to someone that's going through something tremendously difficult*', W5, adv1),<br>• Facilitating shared treatment decision-making '*by breaking up with the medical view that is out there where they are on top and make the decision and decide what information they give us or not, when the body and health are ours*' (W2, Pa1),<br>• Decreasing feelings of isolation ('*I think it helps reduce the loneliness that can be felt by someone who is in this situation, realising that other people have the same questions, and having the answers there, I think it is undoubtedly a very, very good support*', W3, Pa1),<br>• Increasing access to psychosocial support during and particularly after unsuccessful treatment. Patients considered the digital educational resources '*would be part of the support from clinics (…) like half of what the clinic is responsible for providing, which would be the psychological accompaniment*' (W1, Pa1),<br><br><u>Perceived barriers do not outweigh the benefits</u>:<br>• Patients acknowledged some adverse effects of the educational resources, such as triggering negative emotions or crushing patients' optimism towards treatment ('*you are really very excited and maybe seeing these materials … is like you get stuck and go back to zero again*', W2, Pa3) and jeopardising their engagement with treatment, as '*looking at it from the final goal that is to get pregnant, maybe someone could make the decision not to even start treatment, that you scared someone with so much reality, basically*' (W1, Pa5). However, patients also believed that, as the educational resources include '*signposting links and contacts they [patients] can seek for further advice or support*' (W5, Pa5), patients would find it supportive and comforting ('*I think that if this type of information reached everyone on time, even if it's cruel, it would avoid a lot of pain*', W2, Pa9).
**Category: digital educational resources strike the right tone**	• All staff considered the digital educational resources very '*thorough*' (W1, CL1), '*attractive*' (W1, N1), '*helpful*' (W2, Psych2), and '*very useful, mainly because we don't have, and the patients don't have any alternatives*' (W1, Psych4).<br>• Staff valued that '*everybody can freely access [the educational resources], and that is freely advertised*' (W1, CL1). Regarding the content, staff expressed positive views towards specific sections of the resources. On the clinics' web page, staff valued having '*practical tools*' (W1, Psych1) to promote preventive end-of-treatment care provision, particularly the common questions and concerns section, which would help them be aware of patients' preferences and needs. On the patients' page, staff valued '*the mixed media. I like that you have the video and then you have the written part*' (W1, N1), and in particular, the video animation: '*how it's executed*' (W1, CL1), considering it '*very schematic*' (W1, CL1), '*suggestive*' (W1, Psych1), and '*leaving a lot to the imagination*' (W1, CL1).	'*It's comforting (…) it's like a virtual hand, isn't it!?*' (W3, Pa2)<br>• Providing '*reliable information*' (W3, Pa1) all '*under the same roof*' (W4, Pa1). Patients referred that '*the resources have loads of great points to it*' (W5, adv1), providing '*support, information, guidance, points for reflection, ways that you can try and progress and move forward*' (W5, Pa5).<br>• All patients expressed positive views towards the digital educational resources, considering them '*really good*' (W4, adv2), '*super-interesting*' (W2, adv2) and '*super useful*' (W2, Pa14). All were highly willing to engage with the resources, referring to them as '*totally necessary*' (W2, Pa9) ('*I really needed something like that, some support like that (…) it's very valuable for the patients*', W1, Pa1),<br>• Most patients referred that they '*don't have any negative views because I think it's a very (…) I think a lot of thought has been put into, you know, the visual and, of course, the content, you know, itself, I think it's just a really, really positive tool*' (W4, adv2),<br>• Patients appreciated the features of the resources being online and easily accessible—'*updated to our times*' (W3, Pa1), '*open and free*' (W1, Pa1)—

(continued)

Supplementary Table S3. Continued

	Fertility staff (n = 15)	Patients and patient advocates (n = 41)
		anonymous and self-contained, and also valued having both written and video information,<br>• Regarding the content, patients particularly valued the video and the common questions and concerns section (‘*the questions [common questions and concerns] and the video very, very well done*’, W1, Pa5; ‘*absolutely brilliant [questions and concerns section]*’, W4, adv2), considering that overall the resources convey a realistic approach to the topic (‘*I like kind of the imagery that it doesn’t have babies because I notice a lot of things about infertility will include baby pictures and pregnant people all over it*’, W5, Pa2),<br>• ‘*It just has the right tone, right colours, you know, the content looks comprehensive, you know, the questions (…) I think it looks really appealing, and I do think it would be well received*’ (W4, adv2).
**Category: clinics benefit from signposting patients and staff to resources**	<u>When</u>:<br>• Most staff considered the educational resources ‘*should come later*’ (W1, N2, Psych3) in the treatment pathway, with some suggesting the ‘*cycle review appointment*’ (W1, Psych2) after at least one cycle ‘*had completely failed*’ (W1, N1), as patients have already experienced a failed cycle and can ‘*relate*’ (W1, N1) with it. Giving access to resources later could also ‘*ensure better adherence to whatever the clinic offers as a follow-up care or follow-up plan*’ (W1, CL1). Staff considered that exploring the resources ‘*at the beginning is a little bit scary (…) too discouraging*’ (W1, N1), could ‘*scare patients away*’ (W1, CL1), and negatively impact patients’ trust in the clinic. However, staff also agreed that ‘*at the end of the road (…) it’s not the right time either*’ (W2, CL1).<br><u>Dissemination</u>:<br>• Staff agreed they ‘*would always mention, I would mention this [the educational resources] as part of the road they [patients] have to travel but (…) I would not put pressure on it*’, W2, CL1),<br>• Staff highlighted the resources should be disseminated, in particular among staff, to ‘*train staff, broadening their view*’ (W2, CL2) and to ‘*try to make it [preventive end-of-treatment care] a routine*’ (W2, Psych 2) practice at clinics, as ‘*the more your website gets out there, then the more staff members know about it, and even the counsellors and psychologists are referring [patients] to it*’ (W1, Psych2),<br>• In general, staff expressed concerns about disseminating the resources among patients. Some considered it could be made available on the clinic’s website ‘*but not on the front page, it will be down at the bottom, somewhere*’ (W1, N1), ‘*like additional information*’ (W1, CL1), due to the same reasons expressed above. They also considered that ‘*patient associations are a good place to post it because patients who get desperate and are thinking:—“should I go to another clinic, yes or no, what is my way out?,” I mean, they might go there too, and I am doubting whether they’re coming to us, right?*’ (W1, N1).	<u>When</u>:<br>• Almost all patients would like to be signposted to the educational resources ‘*at the beginning, whether the treatment is low or high complexity*’ (W1, Pa4), with some additionally stressing they would like to be given the possibility of revisiting the resources during the journey (‘*it is good to have them there accompanying us (…) after each negative outcome it is good to remember and refresh that feeling that we are supported*’, W2, Pa14),<br>• Two patients may would like to be signposted to the resources and have more in-depth discussions with staff after at least one unsuccessful cycle because ‘*at another time, it could be a hard blow*’ (W5, Pa3),<br>• Some patients recognized ‘*when they [patients] want to receive information and what information they want to receive*’ (W2, adv2) might ‘*depend on each person*’ (W2, adv1), but that it ‘*should be part of the initial treatment (…) and always offered to patients*’ (W1, Pa1). Patients agreed that staff should exercise discretion on the depth with which they approach the possibility of unsuccessful treatment, according to the patient’s preferences: ‘*I think personalisation is important. Some people just don’t want to think about that [unsuccessful treatment], and that’s ok, but to know that it’s there and have it as a resource. I don’t think it could ever be negative*’ (W5, Pa2).<br><u>Dissemination</u>:<br>• Patients agreed that the digital educational resources (in particular the patients’ video) should be offered empathically and in-person by the clinic, in particular by the clinician, as they are ‘*the closest person to us*’ (W3, Pa2) and ‘*the most trusted person*’ (W3, Pa1) or the mental healthcare professional. It should also be made available online and disseminated as much as possible. Patients referred clinics should embed the resources on their websites, with many patients highlighting they would be much more likely to choose that clinic if this information were there (‘*I would say that this clinic would immediately go up a few points in my consideration (…) [It] would demonstrate the clinic or the hospital is concerned with the emotional part of the treatment*’, W3, Pa1,—‘*Me too*’, W3, Pa4; ‘*it would give me confidence. Even if something went wrong, they would be right there for me*’, W3, Pa4). Notwithstanding, they stressed they would feel ‘*absolutely devastated if they [staff] didn’t follow through on that promise of advice and support*’ (W5, Pa2).
**Category: more content and features can be added to improve**	Staff provided further suggestions to improve the acceptability and feasibility of the digital educational resources:	Patients made several suggestions to improve the acceptability and feasibility of the digital educational resources:

(continued)

Supplementary Table S3. Continued

	**Fertility staff (n = 15)**	**Patients and patient advocates (n = 41)**
**acceptability and feasibility: emotional and coping resources, support links and testimonies, higher personalization, and tailoring to minoritized groups**	Staff's page<br>• Tailoring treatment success rates according to the type of treatment (i.e. with or donated gametes/ embryos) and number of cycles ('*the data you presented don't necessarily reflect the success of treatment in other countries (…) not all patients have six IVF cycles*', W1, CL1),<br>• Clearly defining what unsuccessful treatment is: '*so this is ending treatment which own gametes, right? (…) So, I think that's an important thing to clarify*' (W1, N1),<br>• Making clearer what the MyJourney web app is '*because I think the reason why many clinics don't have that conversation early enough is because they don't have anything to really offer*' (W1, N1),<br>• One staff questioned if it would make sense to tailor the resources to the staff's background ('*differentiate between clinicians, psychologists and other staff*', W2, CL2).<br>Short video animation<br>• Wording more empathically ('*I do like the video, and I think it's really good. And I think it portrays well, you know, particularly that bit in the beginning, going through the black tunnel and everything. But I think sometimes some of the wording lacks empathy for the patients who are ending treatment. So, it's not necessarily conveying empathy of how rubbish the patient is likely to be feeling*', W1, Psych2),<br>• Clearly differentiating between the digital educational resources and the MyJourney web app: ('*this [the digital educational resources] might be an independent tool because for some patients it might be enough to have all these normalisations of what they are feeling or thinking*', W1, Psych3).<br>Patients' page<br>• Making clearer for whom the digital educational resources are directed and at which stage: '*does it also work for patients who are deciding whether or not to stop? Because I think it's very difficult for patients to decide, that's what I see in my research, that they're really stopping, right?*' (W1, N1);<br>• More containment and measured approach to hope when talking about having alternative fulfilling pathways, mainly a child-free lifestyle, as '*what I find a little bit trouble when you say that unsuccessful IVF patients can have a happy and fulfilling life. Because, you know (…) I have never seen really truly happy unsuccessful IVF patients later on in life*' (W1, CL1),<br>• One staff suggested '*adapting to different cultural backgrounds because I know that in some countries or some subcultures, in western countries as well, there can be a huge stigma on infertility and childlessness*' (W2, ETH1).	Staff's page<br>• Emphasising the importance of staff setting realistic expectations about treatment outcomes and using a measured approach to hope,<br>• Emphasizing that patients feel extremely lonely during and after unsuccessful treatment ('*professionals should know that isolation can be really acute—point out more*', W5, adv1),<br>• Some patients suggested having '*a video designed for the medical team on how to communicate this information*' (W2, adv2).<br>Short video animation<br>• Worded more empathically, with some suggesting soft music in the background: '*the tone has to be a little more friendly (…) also maybe perhaps a little soft music (…) I think that also hugs you sometimes. When you hear a kind voice and beautiful music, it still comforts you*' (W1, adv1),<br>• Some patients '*do wonder a little bit about the train metaphor on the journey*' (W5, Pa2), suggesting it could be softer ('*personally, it struck me as fear, like a feeling of terror rather than as welcoming*', W1, Pa5).<br>Patients' page<br>• Wording directly to the person ('*it is more friendly when (…) you feel that it is directed more at you (…) when it's written directly in the second person*', W1, Pa4),<br>• More containment and measured approach to hope when talking about setting a Plan B and having alternative fulfilling pathways, in particular, a child-free lifestyle ('*I feel that this is something, for me, it is something that impacts me. It is something very delicate (…) very personal*', W1, Pa2),<br>• Having a section with other paths to and beyond parenthood ('*it occurred to me to add this, I think someone said it, that thinking of a life without children is still thinking of a different family configuration. Just as we see here, there are a bunch of people who have the desire to set up a different type of family—all different, and there can also be other family forms even without children*', W2, adv2) and different types of support sources, such as patients' associations, group and peer support, webinars, books, help lines and psychological support ('*peer support is one of the most important things when it comes to dealing with the feelings, with the grief, with the isolation*', WG4, adv2)—one of the most mentioned suggestions,<br>• Including testimonies/personal stories of other patients who have positively moved on from treatment ('*It would be interesting to put a real case, some interview, some family that has failed and tells their experience or how they achieved that… I mean, to be able to see that there is light after the tunnel and beyond the data. That maybe, I mean, it would help me or give me a little more hope from the failure*', W2, Pa13),<br>• Coping strategies tailored to the treatment stage ('*because it goes hand in hand with the moment you are in treatment*', W1, adv1) and specific life events: holidays, menopause ('*I wonder if there is evidence then women do really feel better after menopause? We heard from someone who has fully moved on from treatment and gone through menopause, and they talked about the sadness of their friends becoming grandparents*', W5, Pa2),<br>• Tailoring the resources to others, such as men ('*as the text may perpetuate gender stereotypes*', W5, Pa2), single women and same-sex couples ('*also recognising same-sex couples, that just like heterosexual couples, we also have the same desire to procreate*', W2, Pa14), and minoritized cultures ('*some cultures look*

(continued)

Supplementary Table S3. Continued

	Fertility staff (n = 15)	Patients and patient advocates (n = 41)
		at things very differently obviously (…) Sometimes talking about fertility can be, you know, often not spoken by stigma so that might be helpful for other people looking from different cultural perspectives', W5, Pa3),<br>• A minority suggested presenting the common questions and concerns section in a more '*iconographic*', W1, Pa4) way and with audio files ('*could there also be a person narrating? I kind of imagine a person as being very empathic, very close, very warm, who can kind of say themselves, not written, like it would feel closer perhaps (…) and not have to read because basically, it is just as difficult*', W1, Pa3),<br>• Two patients expressed ambivalent views about using the Journey word: '*I like the journey word, but sometimes I get frustrated with the journey word that everybody uses because to me journeys are usually a fun thing, and now it's like: well now I hate journeys because of this whole experience*' (W5, Pa2).

W, workshop; Pa, patient; Adv, patient advocate; CL, clinician; N, nurse/midwife; Psych, psychologist; EMB, embryologist/andrologist; CM, clinic manager; ETH, ethicist.

© The Author(s) 2026. Published by Oxford University Press on behalf of European Society of Human Reproduction and Embryology.
This is an Open Access article distributed under the terms of the Creative Commons Attribution License (https://creativecommons.org/licenses/by/4.0/), which permits unrestricted reuse, distribution, and reproduction in any medium, provided the original work is properly cited.
Human Reproduction, 2026, 00, 1–28
https://doi.org/10.1093/humrep/deaf248
Original Article
