# Supplementary material for: A multi-country study to co-design and evaluate digital educational resources to support conversations about ending fertility treatment
Source: Hum Reprod. 2026 Jan 7;41(3):381–93. doi: 10.1093/humrep/deaf248 (PMC13017559; doi:10.1093/humrep/deaf248)
Supplement: deaf248_Supplementary_Data_File_S1 [file deaf248_supplementary_data_file_s1.pdf]

Innovation for All award

Extending the international and clinical reach of MyJourney: an online self-help intervention for people with an unfulfilled wish for children.

Cardiff Fertility Studies Research Group
School of Psychology, Cardiff University, UK

School of Psychology

CARDIFF UNIVERSITY
PRIFYSGOL CAERDYDD

myjourney.pt

If you are struggling to build acceptance of your unfulfilled wish for children, MyJourney can help you to build useful skills.

Deal with difficult thoughts, feelings and situations

Develop new insight into who you are and what you value

Explore new pathways in life, looking towards a hopeful future

MyJourney

Agenda

1. Meet team & introduction to project - 10 min
2. Discussion - conversations about possibility of treatment being unsuccessful - 30 min
3. Presentation of proposal for MyJourney package 5 min
4. Discussion - 15 min

Note: With your permission, we will record this section and transcribe text as ‘note taking’. All comments will be anonymised.

MyJourney

Aims & timeline

- Aims
  1. translate MyJourney to Spanish and German
  2. co-produce a ‘MyJourney package’ to:
     - have conversions about the possibility of treatment being unsuccessful
     - introduce MyJourney to their 'end-of-line' patients

- Duration
  - From October 2021 to October 2020 [launch of MyJourney package]

Activity	Timeline	
**Project and stakeholder setup**	October2021	
**Translate MyJourney to Spanish & German**	October-November2021	
**Prepare MyJourney package concept**	December2021	M1
**Consultation exercise**	January-March2022	
**Co-produce MyJourney package with partners**	April-June2022	M2
**Refine MyJourney package in line with clinic feedback**	July2022	
**Launch in Europe and Latin America**	August2022	M3

Outcomes

- Expected outputs

1. MyJourney translated to Spanish and German

2. Co-production of MyJourney package – IP Cardiff University
   - Cost-free
   - Evidence-based & patient-centred
   - Clinics feel confident to implement (easy to use)

3. Co-authorship in article describing process of adapting MyJourney to clinical setting

Your partnership

- Iterative feedback during co-production of MyJourney package
- Support with staff & patients consultation exercises
  - Dissemination of recruitment adds
  - Running consultations (Spanish, German)
- Champion need for conversations about unsuccessful treatment
- Support dissemination & implementation of MyJourney package

MyJourney package

Staff

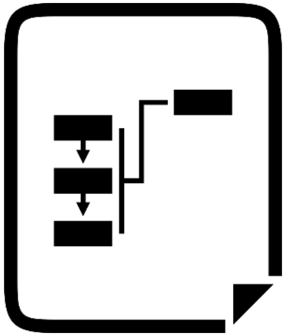

Step by step guidance (online, pdf, paper)

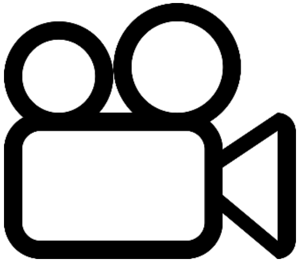

Short video for staff and patients to watch together

Patients

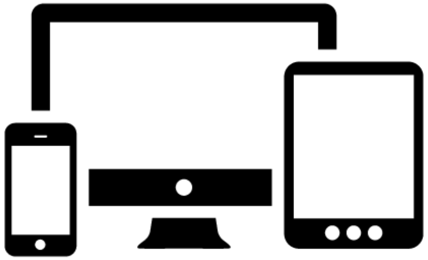

Webpage

Step-by-step guidance

- How to have conversations about support after unsuccessful treatment?

1. Ask patients' permission

2. Watch video together

3. Answer questions and address concerns
   - list of common questions/concerns & possible answers

4. Signpost patients to patient webpage AND/OR within clinic support resources

- Support after unsuccessful fertility treatment

1. Explain what most patients experience and do after unsuccessful treatment
2. Foster hope towards the future (even if undesired)
3. Foster collaborative relationship with fertility team
4. Signpost to webpage/MyMourney (includes vast array of other support resources)

Webpage

- Support after unsuccessful fertility treatment

1. Why it can be helpful to reflect about a "Plan B"
2. Video
3. Frequently Asked Questions - FAQs
   - Similar to list of common questions/concerns [staff guidance] but more in-depth
4. Support resources
