# Supplementary material for: A multi-country study to co-design and evaluate digital educational resources to support conversations about ending fertility treatment
Source: Hum Reprod. 2026 Jan 7;41(3):381–93. doi: 10.1093/humrep/deaf248 (PMC13017559; doi:10.1093/humrep/deaf248)
Supplement: deaf248_Supplementary_Data_File_S2 [file deaf248_supplementary_data_file_s2.docx]

**Supplementary Data File S2**

**Focus Group Script (English version)**

**Introduction**

As you should have read on the information sheet and informed consent, this discussion will be recorded, both video and audio taped. This is for data analysis purposes, and this data will only be shared with members of the research team. Once the discussions have been transcribed, the video and audio files will be permanently deleted, and all data will be anonymised. Therefore, if any ‘word-for-word’ quotes are used in the published results, then efforts will have been made, so you will not be able to identify who said that. Given the topic of today’s discussion, it is possible that, at some points, you may feel upset or uncomfortable. In the end, we forward you links to online support resources, so you can access them if you feel you need to. We will also email you a debrief form, where you can find and access several support services and resources. The debrief form will also have our contact, and we encourage you to contact us if you have any concerns and wish to speak to one of us. You are, of course, free to withdraw at any point in time today from this Zoom discussion. If this happens, we will contact you after as a duty of care to check that you are alright. Does anyone have any questions or concerns about any of this?

Before we get started, I would just like to go over the code of conduct that everyone should adhere to today so we can have a discussion that is as balanced and respectful as possible. Everyone is entitled to their own opinions, views and ideas based on their own personal and professional (for satff) experiences. It is fine if you wish to agree or disagree with what someone else is saying, but please do so as respectfully, non-judgmentally and compassionately as possible. We also ask that you try not to interrupt someone else when they are speaking and wait for your turn to say something. Please feel free to use the virtual hand function. You don’t have to say anything if you don’t want to, but we are very keen to hear the views of everyone attending today; everyone’s contributions are equally important. If you don’t understand a question or need clarity on something, then please do not hesitate to ask. Please also try to focus on the specific topic under discussion, as time is limited. We understand everyone has their own experiences, but please consider which are directly relevant to the discussion. The discussion will last at most 1h30 (for patients)/1h (for staff), so we should be finished by XX. As this discussion is time-limited, there may be points where we must move on to the section or question. If this discussion ends and you still feel that you have more to say, then please feel free to email us with additional written comments after the discussion today has ended.

First, we will ask you about your views and experiences of conversations you have had with healthcare professionals (for patients) / patients (for staff) about fertility treatment being unsuccessful. When we refer to unsuccessful treatment, we mean when all attempted cycles were not successful, and no new cycles will be attempted. We recognise that patients and staff do not always know if patients have reached this point, but we would like to hear from you about when and how you started thinking this might have been a possibility.

After, we will be presenting a proposal for support resources for patients and staff to facilitate these conversations and support patients through this experience. We will be asking for your feedback on these developed resources.

Finally, before we end, you will be debriefed, as previously mentioned.

**Section 1 - Proposal questions on preventive end-of-treatment care provision at clinics**

Focusing on your experiences and views of unsuccessful treatment:

1. What are your views and experiences about how clinics currently support patients for

unsuccessful treatment before, during and after treatment?

- 1. Prompts: If you have personal experience, please share. What could clinics do to improve this support? What things would you need from your clinic/healthcare team to better cope with unsuccessful treatment? (only for patients)

1. Do you think having the opportunity to discuss and prepare in advance for the possibility of treatment being unsuccessful is helpful?
   1. Prompts: Why? When and in what circumstances would it make sense to have these discussions? What could be helpful about these discussions?
2. What would make it easier to have these conversations?
3. Are there any situations or valid reasons NOT to have these conversations?
   1. Prompts: Please explain why.

**Section 2 - Proposal questions on the digital educational resources**

Please note that all these Resources you are about to see are initial proposals, and our aim is to collect feedback from you so that we then develop the final versions that, hopefully, will better meet your needs. We appreciate all types of feedback. Everything you can tell us will be helpful, so feel free to express both positive and negative views.

Presenting participants with the initial prototypes of the digital resources produced

1. What are your views and thoughts about the resources we presented?
   1. Prompts: What did you like most? What did you like the least?
2. Do you think these resources fit the fertility clinics’ organisational culture?
3. Do you think these resources can create any benefits?
   1. Prompts: for patients, for healthcare professionals, or clinics?
4. Would you expect any negative effects from using these resources?
   1. Prompts: for patients, for healthcare professionals, or clinics? Are there any other approaches you think would be more helpful?

**Video animation**

1. What are your views on the video?
   1. Prompts: How can we improve it?
2. How would you react if your clinic invited you to watch this video? (for patients) How do you think patients would react if their clinic invited them to watch this video? (for staff)
   1. Prompts: Positive and negative thoughts and feelings it may trigger? How (and when) would you like to be invited to watch it? (for patients) How (and when) do you think patients would like to be invited to watch it? (for staff) What would you think if this video on your clinic’s webpage?

**Webpage for patients**

1. What are your views about this webpage?
   1. Prompts: How can it be improved? Is there anything missing?
2. Would you explore this webpage on your own? (for patients) Do you think patients would explore this webpage on their own? (for staff)
   1. Prompts: If no, what do you think is stopping you? (for patients). If no, what do you think is stopping patients? (for staff)

**Webpage for staff**

1. What are your views about this webpage?
   1. Prompts: How can it be improved? Is there anything missing?
2. Do you think these resources would encourage healthcare professionals to have discussions about unsuccessful treatment with their patients?
   1. Prompts: What would need to be improved (in case of negative views).

**Closure and debrief**

It is now approaching the finishing time; therefore, we are going to end the discussion here. Does anyone have any brief final comments or questions?

I would like to thank you again for participating. We really appreciate your time and contributions to this important research. As mentioned, you will each be emailed the debrief information soon after this Zoom session ends, with details of how your data will be processed and made available, as well as the team’s contact details, should you have any questions. The debriefing document will also have links to support services and resources, but I will also put links into the chat box to support resources now should you wish to access them right now.
