# Supplementary material for: A multi-country study to co-design and evaluate digital educational resources to support conversations about ending fertility treatment
Source: Hum Reprod. 2026 Jan 7;41(3):381–93. doi: 10.1093/humrep/deaf248 (PMC13017559; doi:10.1093/humrep/deaf248)
Supplement: deaf248_Supplementary_Data_File_S3 [file deaf248_supplementary_data_file_s3.pdf]

Innovation for All award

Extending the international and clinical reach of MyJourney: an online self-help intervention for people with an unfulfilled wish for children.

Cardiff Fertility Studies Research Group
School of Psychology, Cardiff University, UK

School of Psychology

CARDIFF UNIVERSITY
PRIFYSGOL CAERDYDD

myjourney.pt

If you are struggling to build acceptance of your unfulfilled wish for children, MyJourney can help you to build useful skills.

Deal with difficult thoughts, feelings and situations

Develop new insight into who you are and what you value

Explore new pathways in life, looking towards a hopeful future

MyJourney package

Staff

Patients

Guidance **for staff**
(online, pdf, paper)

Short video **for patients**

Webpage **for patients**

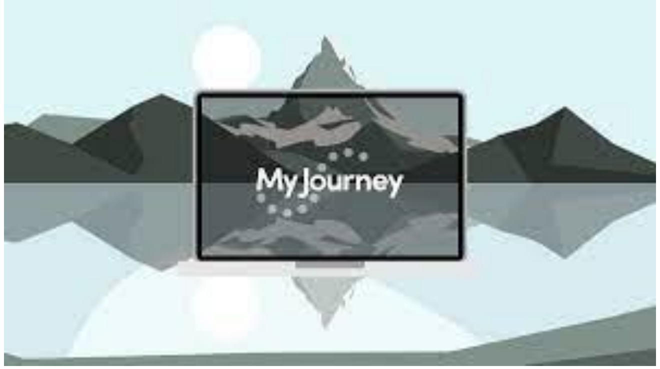

- <u>WHY?</u>

  - For every 10 patients doing three cycles of IVF/ICSI, four will not achieve a live birth. Even when patients do 6 cycles of IVF/ICSI, 2 to 3 out of 10 will not achieve a live birth.
  - Unsuccessful treatment is an extremely difficult outcome for patients. It triggers intense sadness and grief reactions, feelings of guilt, low self-worth and anger. Patients who undergo unsuccessful treatment report moderate to large impairments in their mental-health and wellbeing and take around 2 years to adjust to this outcome.
  - People who use MyJourney, an online self-guided support tool to help build acceptance of one's unfulfilled with for children, experience clinically significant improvements in wellbeing within ten weeks.
  - Regulators and evidence-based guidelines recommend clinics to support patients while they are doing treatment AND when this is unsuccessful.
  - Nine in every ten patients want to discuss the possibility of treatment being unsuccessful while doing treatment.

- <u>WHEN?</u>

Only you can decide if and when you should discuss the possibility of treatment being unsuccessful with each of your patients. Being aware of patients' preferences may help you decide:

- Seven in ten patients want to have this discussion before they start their first IVF/ICSI cycle.
- Three in ten patients only want to have this discussion after they have done at least one unsuccessful IVF/ICSI cycle.
- Five in ten patients report the following valid reasons to have this discussion: bad prognosis, distress during treatment, difficulty accepting the possibility of a negative outcome, and doubts about whether to continue treatment.
- Most patients want to be offered support immediately after the end of unsuccessful treatment, even though some may need time for their grief to decrease before they feel ready to engage with it.

• HOW?

If you choose to discuss the possibility of treatment being unsuccessful **while your patients are still doing treatment**:

- Adopt an empathic and collaborative attitude
- Acknowledge you would like to discuss a sensitive topic
- Inform treatment is not always successful but the clinic will support them in any eventuality.
- Signpost to www.myjourney.pt/patients **[section ending treatment]**. Tell patients they can explore these resources at their own pace and according to their needs and preferences.
- Make yourself available to answer any questions and address concerns. The table below can help you address the most common questions and concerns patients have.

If you choose to contact your patients **after treatment finishes unsuccessfully**:

- Adopt an empathic and collaborative attitude
- Acknowledge treatment was unsuccessful and validating patients' feelings
- Reassure patients there was nothing else they could have done for treatment to work
- Inform patients the clinic is there to support them. Ask if they would like in-person support and make the necessary referral
- Signpost to www.myjourney.pt/patients **[section moving forward after ending treatment]**. Tell patients they can explore these resources at their own pace and according to their needs and preferences.
- Make yourself available to answer any questions and address concerns. The table below can help you address the most common questions and concerns patients have.

• ANSWERING QUESTIONS AND ADDRESSING CONCERNS

Research shows patients have specific questions and concerns they want to discuss. The table below lists these questions and concerns and offers suggestions for answers.

1. How to cope with the difficult thoughts and emotions patients think they will experience, if treatment is unsuccessful

Inform patients that:

- most people go through a grief period after, and it is normal and acceptable if they experience sadness, guilt, low self-worth and anger
- these are normal reactions to loss, not a sign of weakness
- these reactions ease with time and most people learn to live with them
- even if they don't fully disappear, they do not stop people from living a fulfilling life.

Signpost patients to www.myJourney.pt. Tell patients the first three steps of MyJourney invite patients to practice skills known to help people feel better through difficult times. In particular, they help patients to:

- recognise when difficult emotions and thoughts occur and how they feel them in their body
- be more self-compassionate or, in other words, to give themselves the same kindness and care they would give to a friend in trouble
- create distance between themselves and their unhelpful or distressful thoughts.

2. Where and how to access support resources to use in case treatment is unsuccessful

3. What are useful strategies to cope with unsuccessful treatment

4. Discuss the pros and cons of continuing versus ending treatment

5. Examine personal resources to cope with unsuccessful treatment

Invite patients to think about:

- what activities give them pleasure and fulfilled (e.g., hobbies, spirituality, nutrition, exercise)
- who best supports them when they are in need (e.g., loves and understand them, gives helpful advice and practical support)
- what strategies helped them coping with difficult situations they faced in the past
- what things they tell themselves that help them keep an optimistic view about things.

Ask patients if any of the things they referred to could help them cope with unsuccessful treatment.

This webpage

- was designed to support people moving forward when fertility treatment does not work
- is for anyone who has gone through this event or wants to prepare in advance for this possibility
- was informed by the personal experiences, views and needs of many people who went through this event
- provides answers for the most common concerns people have and support for the challenges they may face

Planning is part of a journey

Thinking ahead about how to move forward from unsuccessful treatment may be challenging. However, planning will make your journey easier, and help you set new paths you may want to explore in pursue things you value in life.

It may be helpful to know what you are likely to experience if and when you venture onto a new path

It may be helpful to find answers for concerns you have now about how your future may look like

It may be helpful to learn about available support sources and ways of coping with the challenges you may face

- FAQ: ENDING TREATMENT

1. **How can I decide whether to continue or finish treatment?**

The decision to finish fertility treatment is deeply personal. Below we present information and suggestions that you may find helpful.

Most patients adjust well to ending treatment when they

- feel they were well advised by their fertility team
- understand why previous cycles did not work
- feel they have done ‘all they could’ and/or explored all options available
- do not experience pressure to have children

You can discuss the following topics with your fertility team:

- reasons for why your previous cycles did not work
- (…)

2. **How may I be affected if treatment does not work?**
3. **How may my partnership be affected if treatment does not work?**
4. **Do I have the strength and resources to cope with treatment not working?**
5. **How can I explore other routes to become a parent?**
6. **Can I reach closure from my fertility treatment?**
7. **What can I do now that can help me later on, if treatment does not work?**

- <u>FAQ: MOVING FORWARD AFTER ENDING TREATMENT</u>

1. **How can I cope with the difficult thoughts and emotions ending treatment can trigger?**

It is important to acknowledge that most people go through a grief period after ending treatment. Your experience will most likely be similar. You should know that:

- it is normal and acceptable if you feel sad, guilty, unworthy, or even just angry and frustrated
- these are normal reactions to loss, not a sign of weakness
- these reactions ease with time and most people learn to live with them
- even if they don't fully disappear, they do not stop you from living a fulfilling life.

www.myJourney.pt gives you step-by-step guidance to practice skills known to help people feel better through these difficult times. In particular, it can help you to: (...)

2. **Where can I access support resources?**
3. **What are useful strategies to cope with treatment not working?**
4. **How can I talk to my partner about moving forward as a couple?**
5. **How can I tell others that I have finished treatment?**
6. **How can I talk with other people who lived through treatment not working?**
7. **How can I cope with other people's comments?**
8. **Are there any advantages for having a childfree lifestyle?**

- <u>FAQ: LIVING A FULFILLING AND SATISFYING LIFE</u>

1. **Can I live a fulfilling and satisfying life without the children I wish for?**

Yes, you can! Most people who go through this experience say that it takes time, but in the end you feel

- you are a survivor and that overcoming this challenge makes you grow personally and spiritually
- a sense of freedom from the infertility and treatment experience and hope towards the future
- your strength and agency return and you can better control your life
- a new sense of balance and equilibrium with themselves and in the partnership (when there is one)
- a better sense of what is important for your and ability to appreciate the ‘good things’ in life.

2. **Will the pain of not having the children I desire ever go away?**
3. **How can I let go of my desire for children?**
4. **When will I feel ready to look for other things in life?**
5. **What can I do to help me move forward towards other things in life?**
